# A pan-cancer single-cell transcriptional analysis of antigen-presenting cancer-associated fibroblasts in the tumor microenvironment

**DOI:** 10.3389/fimmu.2024.1372432

**Published:** 2024-06-06

**Authors:** Juntao Chen, Renhui Chen, Jingang Huang

**Affiliations:** ^1^ Shenshan Medical Center, Memorial Hospital of Sun Yat-Sen University, Shanwei, China; ^2^ Department of Otolaryngology-Head and Neck Surgery, Guangdong Provincial Key Laboratory of Malignant Tumor Epigenetics and Gene Regulation, Medical Research Center, Sun Yat-Sen Memorial Hospital, Sun Yat-Sen University, Guangzhou, China; ^3^ Medical Research Center, Guangdong Provincial Key Laboratory of Malignant Tumor Epigenetics and Gene Regulation, Guangdong-Hong Kong Joint Laboratory for RNA Medicine, Sun Yat-Sen Memorial Hospital, Sun Yat-Sen University, Guangzhou, China

**Keywords:** antigen-presenting CAFs, single-cell RNA-seq, cancer-associated fibroblasts, tumor microenvironment, CD4+ effector T cells, C1Q molecules

## Abstract

**Background:**

Cancer-associated fibroblasts (CAFs) are the primary stromal cells found in tumor microenvironment, and display high plasticity and heterogeneity. By using single-cell RNA-seq technology, researchers have identified various subpopulations of CAFs, particularly highlighting a recently identified subpopulation termed antigen-presenting CAFs (apCAFs), which are largely unknown.

**Methods:**

We collected datasets from public databases for 9 different solid tumor types to analyze the role of apCAFs in the tumor microenvironment.

**Results:**

Our data revealed that apCAFs, likely originating mainly from normal fibroblast, are commonly found in different solid tumor types and generally are associated with anti-tumor effects. apCAFs may be associated with the activation of CD4+ effector T cells and potentially promote the survival of CD4+ effector T cells through the expression of C1Q molecules. Moreover, apCAFs exhibited highly enrichment of transcription factors RUNX3 and IKZF1, along with increased glycolytic metabolism.

**Conclusions:**

Taken together, these findings offer novel insights into a deeper understanding of apCAFs and the potential therapeutic implications for apCAFs targeted immunotherapy in cancer.

## 1 Introduction

The tumor microenvironment (TME) is composed of immune cells, fibroblasts, endothelial cells, signaling molecules, and the extracellular matrix, among others (1). Cancer-associated fibroblasts (CAFs) are the predominant stromal cells found in TME, exerting a crucial influence on the biological characteristics of tumor initiation, progression, metastasis, and therapeutic resistance (2). Thus, CAFs play a critical role in shaping the TME through their interactions with other TME components, making them valuable as both prognostic factors and therapeutic targets.

CAFs exhibit significant heterogeneity, with derivation from normal fibroblasts (NFs), pericytes, smooth muscle cells, epithelial cells, endothelial cells, adipocytes, or mesenchymal stem cells (3, 4), followed by transformation by the neoplastic microenvironment. As a result of the heterogeneity among CAFs, the lack of a universal characterization of CAFs hinders CAFs-targeted therapy in clinical settings (5). By employing single-cell transcriptomics analysis, they can be broadly classified into myofibroblastic CAFs (myCAFs), inflammatory CAFs (iCAFs), and antigen-presenting CAFs (apCAFs) (6, 7). Much efforts have been spent on the first two subpopulations, while apCAFs have gradually received more attention in recent years.

Previous studies have unveiled that fibroblasts in normal tissue possess the ability to function as amateur antigen-presenting cells (APCs) through the processing and presentation of antigens. As early as in 1980s, one study revealed that dermal fibroblasts can present the tetanus toxoid antigen to antigen-specific T cells, leading to an increased cell proliferation response caused by the antigens (8). Another study provided evidence demonstrating the efficacy of fibroblasts as APCs in lymphoid organs (9). Moreover, several studies have shown that synovial fibroblasts in arthritis express major histocompatibility complex (MHC) class II molecules and exhibit the capacity to present arthritis propeptides to T cells (10–13). Interestingly, it has been observed that mouse and human fibroblasts, when cultured *in vitro*, have the potential to undergo reprogramming and transform into conventional type 1 dendritic cells (cDC1) like APCs (14, 15).

Within the context of tumor, Elyada E et al. introduced the novel concept that apCAFs are present in pancreatic ductal adenocarcinoma (PDAC) (6). Subsequent study unveiled that apCAFs are actively engaged in promoting the differentiation of naive CD4+ T cells into regulatory T cells (Tregs) in PDAC (16), thus exerting immunosuppressive role. However, recent research by the Kerdidani D et al. revealed the presence of immunostimulatory apCAFs in non-small-cell lung cancer (NSCLC), which activated and promoted survival of anti-tumor CD4+ effector T cells (17), which was highlighted by Dr. Anna Dart, the editor of Nature Reviews Cancer. Subsequently, Tsoumakidou M introduced the “2nd hit hypothesis” suggesting that anti-tumor T cells may require an *in situ* interaction with apCAFs within the tumor tissue to effectively unleash their anti-tumor capabilities (18). These findings highlight the recognition of the apCAFs concept and the significance of antigen presentation, establishing it as a novel research area. However, it is largely unknown about the existence of apCAFs in other solid tumor types, their cellular origin, the molecular mechanisms governing their formation, and the intricate molecule machinery involved in antigen processing and presentation.

In this study, we collected datasets of 9 different solid tumor types from the Gene Expression Omnibus (GEO) database and the Genomic Data Commons (GDC) database. By utilizing single-cell transcriptomics, spatial transcriptomics, and other bioinformatics analyses, we found that apCAFs were identified in different solid tumor types. Moreover, apCAFs are associated with anti-tumor effects across most solid tumor types and enriched in immune response pathways related to T cells activation, antigen processing and presentation, as well as response to interferon, and the classical molecular components of antigen processing and presentation are highly expressed in apCAFs. apCAFs may mainly come from tissue resident fibroblasts, the transcription factors RUNX3 and IKZF1 may drive its formation. These findings have the potential to provide deeper understanding of apCAFs and novel insights into anti-tumor immunotherapy.

## 2 Materials and methods

### 2.1 Single-cell RNA-seq datasets collected in this study

We gathered single-cell RNA sequencing (scRNA-seq) data from 210 samples, encompassing the 9 common solid tumor types. These samples were obtained from previously published studies. By means of the gganatogram R package (Version 1.1.1) (19), we created a human anatomy schematic to visualize the tissue sources of the scRNA-seq data collected from various solid tumor types. Processing of the raw FASTQ files from the 10× Genomics platform was conducted using CellRanger (version 6.1.2) (20). To start, the count function of CellRanger was employed to align and assess gene expression levels in individual cell, utilizing barcode recognition and unique molecular identifier (UMI) counting. Subsequently, the outputs were transformed into a Seurat object using the Seurat R package (version 4.2.1) (21–24) for further analyses. To consolidate the dataset, we utilized the merge function of Seurat to combine multiple Seurat objects into an integrated Seurat object. Next, quality control measures were implemented on the integrated Seurat object. The initial step involved filtering cells, where we retained cells with a minimum of 200 detected genes and genes expressed in at least 3 cells. Next, we excluded cells with unique feature counts exceeding 4000 or falling below 200, as well as those with mitochondrial counts surpassing 10%. Following the removal of unwanted cells, data normalization was performed using the NormalizeData function in Seurat, and highly variable genes were identified within the individual cell. These highly variable features were subsequently employed in downstream analyses. The data was linearly transformed using the ScaleData function of Seurat, and principal component analysis was conducted on the scaled data. Considering that the data originated from different samples, we utilized the Harmony R package (version 0.1.1) (25) to mitigate batch effects and ensure reliable downstream analyses. Moreover, we chose 20 principal components (PCs) to serve as the input for the Uniform Manifold Approximation and Projection (UMAP) algorithm. Importantly, employing the clustree R package (version 0.5.0) (26), we conducted unsupervised hierarchical clustering to attain an appropriate resolution. Subsequently, we used the FindClusters function in Seurat to classify the cells into distinct cell types, referencing classical markers described in published articles. In addition, we employed the FindAllMarkers function of Seurat to identify differentially expressed genes specific to each cell type. Lastly, we further reclustered CAFs population, myeloid cells population, and T cell population at individually optimized resolutions, followed by annotation using classical cell type markers to identify each subpopulation. Additionally, we utilized the apCAFs gene signature along with the CD4+ effector T cells gene signature as gene sets for each type of solid tumor. We computed the apCAFs gene signature scores and CD4+ effector T cells gene signature scores for each cell in the scRNA-seq data of each solid tumor type, employing the AddModuleScore algorithm within the Seurat R package.

### 2.2 Bulk RNA-seq datasets collected in this study

By using the TCGAbiolinks R package (Version 2.26.0) (27, 28), bulk RNA sequencing (RNA-seq) data containing TCGA-HNSC, TCGA-OV, TCGA-CESC, TCGA-COAD, TCGA-READ, TCGA-BRCA, TCGA-SKCM, and TCGA-KIRC datasets from the GDC database were collected. The TCGAbiolinks function GDCquery was employed to retrieve information concerning various tumors from the GDC database. Subsequently, the GDCdownload function of TCGAbiolinks was utilized to download the retrieved results. Moreover, the GDCprepare function of TCGAbiolinks was used to read and organize the downloaded data into an R object. Finally, the TCGAanalyze_Preprocessing function of TCGAbiolinks was applied to preprocess the data and obtain gene expression data. Furthermore, we acquired Nasopharyngeal Carcinoma RNA-seq data GSE102349 from the GEO database.

### 2.3 Obtaining gene signatures of apCAFs for various solid tumor types and human CD4+ effector T cells

In order to obtain the gene signatures of apCAFs for various solid tumor types, we employed the FindMarkers function in Seurat to detect the differentially expressed genes (DEGs) between apCAFs and other CAFs. For significant DEGs selection, we applied filters of log fold change (logFC) > 0.5 and adjusted p-value (adjPval) < 0.05. From these DEGs, we extracted the top 40 genes exhibiting the highest logFC to serve as candidate genes for the apCAFs gene signature. Furthermore, we acquired the gene signature for human CD4+ effector T cells from a previously published article (17).

### 2.4 Deconvolution of Cell Types with CIBERSORTx

CIBERSORTx is employed as a tool for estimating cell type proportions through deconvolution, utilizing bulk gene expression data to infer the distribution of different cell types within a mixed sample (29). The process began with the retrieval of bulk RNA-seq data from the GDC database using the TCGAbiolinks R package. Subsequently, the bulk RNA-seq data matrices (Mixture) underwent normalization to TPM (Transcripts Per Kilobase Million) and were converted to tab-delimited text format to facilitate further analysis. A reference gene expression matrix (Signature Matrix) was then constructed from single-cell gene expression data obtained using the FindAllMarkers function of Seurat for the same solid tumor type. This Signature Matrix comprises gene expression profiles of annotated cell types that we intended to identify in the bulk RNA-seq data. The Signature Matrix was also converted to tab-delimited text format for subsequent analysis. Both the Mixture and Signature Matrix were uploaded to the CIBERSORTx website (https://cibersortx.stanford.edu/), where deconvolution was carried out utilizing the “Impute Cell Fractions” module. For bulk RNA-seq data, it was advisable to disable quantile normalization, while all other CIBERSORTx parameters remained at their default settings. After the execution, we retrieved the output to be used in the analysis for the next steps.

### 2.5 Survival analysis

Previously, the CIBERSORTx algorithm was used to calculate the estimated abundance of apCAFs in bulk RNA-seq data from various solid tumor types obtained from the GDC database and the GEO database. Subsequently, this estimated abundance was merged with the corresponding clinical information to perform a Kaplan-Meier survival analysis using the survival R package (Version 3.4.0). Finally, we utilized the survminer R package (Version 0.4.9) to visualize the resulting survival curve.

### 2.6 Cell type mapping with NNLS in semla

The semla R package (version 1.0.0) (30) serves as a valuable tool for examining and visualizing spatial transcriptome (ST) data, supporting the Seurat object. The semla is an updated alternative to the classic spatial transcriptomics analysis software, STUtility, developed by The Spatial Research Lab. In addition, the tibble R package (version 3.2.1) was used to construct the infoTable, which contains path information for files such as “filtered_feature_bc_matrix.h5”, “tissue_hires_image.png”, “tissue_positions_list.csv”, and “scalefactors_json.json”. Upon reading the specified ST file from the infoTable, the ST Seurat object was formulated through the application of semla’s ReadVisiumData function. Additionally, we imported scRNA-seq data (the SC Seurat object) from the same solid tumor type, which had previously undergone clustering and annotation. Both the ST Seurat object and SC Seurat object were normalized using the NormalizeData function from semla. To directly infer cell type proportions from the expression profiles of the ST Seurat object, the RunNNLS function of semla, based on Non-Negative Least Squares (NNLS), was applied, utilizing the SC Seurat object as a reference. Finally, for visualization purposes, the semla’s MapFeatures and MapMultipleFeatures functions were employed. Moreover, in preparation for the subsequent correlation analysis, we utilized the FetchData function of semla to obtain the cell type proportions for each spot in the tissue slice.

### 2.7 Spatial transcriptomics analysis via Seurat

After loading the matrix files of the ST for each slice, a Seurat object was constructed using the Seurat function CreateSeuratObject. Next, the Seurat function Read10X_Image was used to read the files “tissue_lowres_image.png”, “tissue_hires_image.png”, “tissue_positions_list.csv”, and “scalefactors_json.json”, generating a VisiumV1 object. Then, the VisiumV1 object was integrated into the Seurat object, forming a ST Seurat object for each slice. The created ST Seurat object was ready for subsequent analysis. To begin with, the Seurat function SCTransform was applied to normalize the ST Seurat object. Then, the gene signatures of CD4+ effector T cells and apCAFs of different solid tumor types were read. Next, the Seurat function AddModuleScore was used to calculate gene signature scores for CD4+ effector T cells and apCAFs in each spot on the tissue slice. Subsequently, the Seurat function SpatialFeaturePlot was employed to visualize the distribution of the CD4+ effector T cells gene signature and the apCAFs gene signatures of various cancer types in the tissue slice. Lastly, for the purpose of subsequent correlation analysis, gene signature scores for the CD4+ effector T cells and the apCAFs of different solid tumor types were obtained for each spot within the tissue slice.

### 2.8 GSVA

Employing the GSVA R package (version 1.46.0) (31), we evaluated the molecular phenotypes of individual cell using the raw count matrix of scRNA-seq data. Specifically, we obtained GSVA gene set enrichment scores for HALLMARK and GO: BP gene sets from the msigdbr R package (Version 7.5.1) for individual cell of apCAFs and NFs. Subsequently, we employed the limma R package (Version 3.54.2) (32) to compare pathway variances between apCAFs and NFs. The resulting t-values were visualized as a bar plot using the ggplot2 R package (Version 3.4.2). Furthermore, using the scaled data matrix of scRNA-seq data, we obtained GSVA gene set enrichment scores for HALLMARK gene sets from the msigdbr R package for individual cell of various fibroblasts subpopulations in certain solid tumor types. Subsequently, we utilized the ggboxplot function provided by the ggpubr R package (Version 0.6.0) to visualize the differences in GSVA enrichment scores of glycolysis pathway for each fibroblasts subpopulation. Moreover, using the apCAFs gene signature and CD4+ effector T cells gene signature as gene sets, we applied GSVA to calculate gene signature enrichment scores for each sample of the bulk RNA-seq data from various solid tumor types in the GDC database and the GEO database.

### 2.9 Correlation analysis

The CIBERSORTx algorithm was previously employed to estimate the abundances of distinct cell types in bulk RNA-seq data from various solid tumor types obtained from the GDC database and the GEO database. Furthermore, the GSVA algorithm was previously used to compute apCAFs gene signature and CD4+ effector T cells gene signature enrichment scores for each sample of the bulk RNA-seq data from different solid tumor types in the GDC database and the GEO database. In addition, the FetchData function of semla was previously utilized to calculate the cell type proportions for each spot in the tissue slice. Moreover, gene signature scores for the CD4+ effector T cells and the apCAFs of different solid tumor types were previously obtained for each spot within the tissue slice using the Seurat function AddModuleScore. Lastly, the data obtained above has been used for correlation analysis, respectively. Employing the ggscatter function provided by the ggpubr R package, individual scatter plots were generated for selected two cell types. We utilized the Spearman’s rank correlation coefficient to analyze the correlation between these two cell types.

### 2.10 Similarity analysis

In order to assess the transcriptomic similarity between apCAFs and their potential source cell types, we employed the cor function from the stats R package (Version 4.2.2), including the top 5000 variably expressed genes. The resulting data was visualized using the corrplot R package (Version 0.92).

### 2.11 Trajectory analysis

For exploring the developmental origins of apCAFs, Monocle R package (version 2.22.0) (33–35) was employed to analyze the cellular subtype expression signatures. The Seurat objects representing the cell subtypes were converted into Monocle CellDataSet using the newCellDataSet function. To ensure data quality, the detectGenes function was utilized to filter out cells with low-quality expression profiles. For the identification of signature genes, differential gene expression analysis was conducted using the differentialGeneTest function. Monocle then inferred the differentiation trajectory of apCAFs using default parameters after dimension reduction and cell ordering. For visualization purposes, we utilized the plot_cell_trajectory function and plot_genes_in_pseudotime function from Monocle, along with the ggsci R package (Version 3.0.0). Moreover, to visualize the distribution pattern of various cell subtypes along the assumed time axis, we employed the ggridges R package (Version 0.5.4).

### 2.12 Single-cell regulatory network inference and clustering using pySCENIC

We performed the transcription factors analysis in distinct fibroblasts subpopulations using the pySCENIC python package (version 0.12.1) (36), following the recommended workflow and utilizing raw counts as input. Initially, we inferred the gene co-expression network using the GRNBoost2 algorithm. Subsequently, we predicted enriched motifs associated with gene co-expression modules by leveraging pre-calculated databases from cisTargetDB and the ctx function in pySCENIC. To assess the activity scores of inferred regulons at the single-cell level, we employed the AUCell function of pySCENIC. The resulting output from pySCENIC (loom file) was then subjected to analysis using the SCopeLoomR R package (Version 0.13.0). From the provided loom file, we obtained the AUCell matrix by utilizing the get_regulons_AUC function of SCopeLoomR and subsequently extracted the AUC matrix using the getAUC function from AUCell R packages (Version 1.20.2). To determine the regulon specificity scores for each cell type, we employed the calcRSS function from the SCENIC R package (Version 1.3.1). Lastly, we selected the three most specific regulons for each cell type and visualized their Z-scores of AUC scores using the pheatmap R package (Version 1.0.12).

### 2.13 Single-cell metabolic analysis

scMetabolism is an R package (Version 0.2.1) (37) specifically designed to assess metabolic activity at the single-cell level, and it comes pre-loaded with 85 KEGG pathways and 82 Reactome entries. We employed scMetabolism to evaluate the metabolic activity of each cell within distinct fibroblasts subpopulations across all metabolic pathways. For optimal visualization, we utilized the DotPlot.metabolism function from scMetabolism to depict the differences in selected metabolic-associated pathways.

### 2.14 Single-cell metabolic flux estimation analysis

We extracted the raw count matrix from the Seurat object containing the different fibroblasts subpopulations. Then, we refined this raw count matrix by selecting genes using scFEA.human.genes. The refined raw count matrix was saved in CSV format and uploaded to the FLUXestimator website (http://scflux.org/) for scFEA analysis (38, 39). Using scRNA-seq data, the scFEA analysis utilized a graph neural network model to compute cell-specific metabolic flux, referencing the module gene file and a stoichiometry matrix that delineates the links between compounds and modules. After the scFEA analysis, we obtained predicted metabolic flux results for each cell of each fibroblasts subpopulation. Finally, we utilized the ggboxplot function from the ggpubr R package to visualize the differences in predicted metabolic flux for each fibroblasts subpopulation’s metabolic module.

### 2.15 Statistics

All analyses were performed using R version 4.2.2. Wilcoxon rank sum test was applied to identify statistical differences between the two continuous variable groups, considering a p-value < 0.05 as statistical significance. During the differential gene expression analysis sections conducted with the Seurat R package, the differential pathway expression analysis with the limma R package, the Kaplan-Meier survival analysis using the survival R package, and the transcriptomic similarity analysis performed using the stats R package, p-values were computed using the standard methods inherent to each specific R package. The Wilcoxon signed rank test was performed using the ggpubr R package to compare the differences in signature scores of cell type determined by CIBERSORTx algorithm, as well as the CD4+ effector T cells and apCAFs gene signature scores determined by GSVA R package. Additionally, it was used to assess the enrichment scores of the glycolysis pathway determined by the GSVA R package, the CD4+ effector T cells and apCAFs gene signature scores determined using Seurat’s AddModuleScore function, and the metabolic fluxes estimated by the scFEA algorithm.

## 3 Results

### 3.1 Identification of antigen-presenting CAFs in various solid tumor types

**Figure 1:**
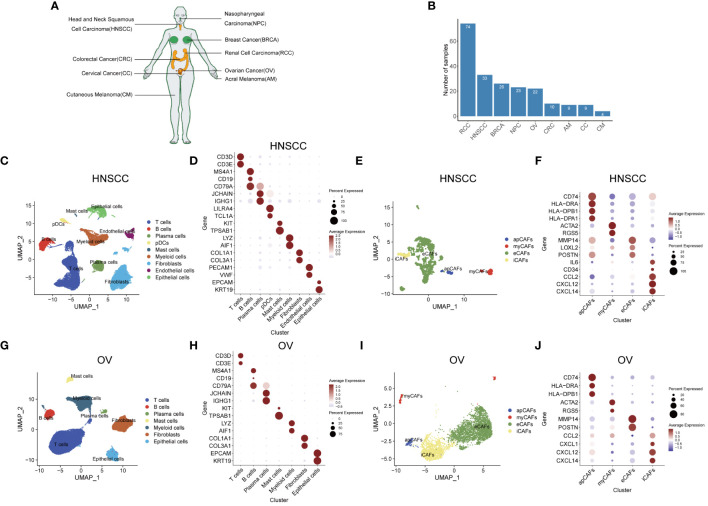
Identification of apCAFs in various solid tumor types. **(A)** All solid tumor types included in this study. **(B)** Bar plot showing the number of samples collected for each solid tumor type. **(C, G)** UMAP plots showing the major cell types in HNSCC **(C)** and OV **(G)**. **(D, H)** Dot plots showing expression levels of selected cell marker genes of the major cell types in HNSCC **(D)** and OV **(H)**. **(E, I)** UMAP plots showing 4 major subpopulations of CAFs in HNSCC **(E)** and OV **(I)**. **(F, J)** Dot plots showing expression levels of selected cell marker genes for the major subpopulations of CAFs in HNSCC **(F)** and OV **(J)**. Dot size indicates fraction of expressing cells, colored based on normalized expression levels **(D, F, H, J)**. HNSCC, Head and Neck Squamous Cell Carcinoma; OV, Ovarian Cancer; NPC, Nasopharyngeal Carcinoma; CC, Cervical Cancer; CRC, Colorectal Cancer; BRCA, Breast Cancer; AM, Acral Melanoma; CM, Cutaneous Melanoma; RCC, Renal Cell Carcinoma; apCAFs, antigen-presenting CAFs; myCAFs, myofibroblastic CAFs; eCAFs, extracellular matrix CAFs; iCAFs, inflammatory CAFs, pDCs, plasmacytoid dendritic cells.

To examine if apCAFs were present in the microenvironment of solid tumors, we collected scRNA-seq data from 9 common solid tumors types containing Head and Neck Squamous Cell Carcinoma (HNSCC), Ovarian Cancer (OV), Nasopharyngeal Carcinoma (NPC), Cervical Cancer (CC), Colorectal Cancer (CRC), Breast Cancer (BRCA), Acral Melanoma (AM), Cutaneous Melanoma (CM), and Renal Cell Carcinoma (RCC) (**Figure 1A**), with 210 samples in 9 studies (**Figure 1B**). In addition, we obtained ST data from 4 solid tumor types, namely HNSCC, OV, BRCA, and CRC (**Supplementary Table 1**). To exclude the possibility of technology-driven bias, we ensured that all scRNA-seq data were consistently generated using the 10× Genomics platform. We employed the Seurat R package and Harmony R package to perform quality control and batch effects removal for each solid tumor type. The cells we retained have unique feature counts below 4000 and above 200, and their mitochondrial counts were maintained below 10%. Quality checks of detected nFeature_RNA, nCount_RNA, percent.mt and batch effects were performed in each solid tumor type (**Supplementary Figures 1A-I**; **2A-I**).

After performing quality control and rectifying batch effects, we undertook unsupervised hierarchical clustering of the overall single-cell transcriptomic profiles encompassing various solid tumor types, with the aim of selecting an appropriate resolution (**Supplementary Figures 3A-I**). We then selected a resolution of 0.3 for HNSCC, 0.2 for OV, 0.2 in NPC, 0.3 for CC, 0.1 for CRC, 0.1 for BRCA, 0.1 for AM, 0.2 for CM, and 0.1 for RCC. Similarly, we conducted unsupervised hierarchical clustering on the comprehensive single-cell transcriptomic profiles of CAFs derived from different solid tumor types, aiming to choose an optimal resolution (**Supplementary Figures 4A-I**). Following are individual resolution: 0.1 for HNSCC CAFs, 0.2 for OV CAFs, 0.1 for NPC CAFs, 1 for CC CAFs, 0.2 for CRC CAFs, 0.2 for BRCA CAFs, 0.1 for AM CAFs, 0.4 for CM CAFs, and 0.3 for RCC CAFs.

After selecting an appropriate resolution, we employed the Seurat R package to perform principal component analysis and graph-based clustering methods to classify individual cell into different clusters. We captured the transcriptomes of 9 major cell types according to the expression of canonical gene markers and then used the UMAP algorithm to reduce the dimensionality and visualize the cell distribution in HNSCC (**Figure 1C**). These cells included T cells (CD3D+, CD3E+), B cells (MS4A1+, CD19+, CD79A+), plasma cells (CD79A+, JCHAIN+, IGHG1+), plasmacytoid dendritic cells (pDCs) (LILRA4+, TCL1A+), mast cells (KIT+, TPSAB1+), myeloid cells (LYZ+, AIF1+), fibroblasts (COL1A1+, COL3A1+), endothelial cells (PECAM1+, VWF+) and epithelial cells (EPCAM+, KRT19+) (**Figure 1D**; **Supplementary Table 2**). Given the functional heterogeneity of CAFs in solid tumors, we reclassified CAFs population into different subpopulations using a graph-based clustering approach. 4 types of CAFs subpopulation were annotated in HNSCC with classic markers described in previous studies (40–43) as shown in the UMAP plot. (**Figure 1E**). These CAFs were termed apCAFs, myCAFs, extracellular matrix CAFs (eCAFs) and iCAFs, which were characterized by specific high expression of MHC class II molecules, ACTA2/RGS5 myofibroblastic molecules, MMP14/LOXL2 extracellular matrix molecules, and inflammatory molecules, respectively (**Figure 1F**; **Supplementary Table 3**). Likewise, similar analysis procedure was performed in OV and 7 major cell types consisting of T cells (CD3D+, CD3E+), B cells (MS4A1+, CD19+, CD79A+), plasma cells (CD79A+, JCHAIN+, IGHG1+), mast cells (KIT+, TPSAB1+), myeloid cells (LYZ+, AIF1+), fibroblasts (COL1A1+, COL3A1+), and epithelial cells (EPCAM+, KRT19+) were annotated and shown as the UMAP plot and dot plot (**Figures 1G, H**; **Supplementary Table 2**). Similarly, by utilizing classic markers, we found 4 distinct CAFs subpopulations within OV (**Figure 1I**). These CAFs subpopulations, including apCAFs, myCAFs, eCAFs, and iCAFs, exhibited specific high expression of MHC class II molecules, ACTA2/RGS5 myofibroblastic molecules, MMP14/POSTN extracellular matrix molecules, and inflammatory molecules, respectively (**Figure 1J**; **Supplementary Table 3**). In addition, by employing the same approach and strategy, we could also identify similar major cell types in other 7 types of solid tumor containing NPC, CC, CRC, BRCA, AM, CM, and RCC (**Supplementary Figures 5A, C, E, G, I, K, M**; **Supplementary Table 2**). In addition, we could also distinguish similar CAFs subpopulations within the CAFs population of these 7 types of solid tumor (**Supplementary Figures 5B, D, F, H, J, L, N**; **Supplementary Table 3**). In these 9 types of solid tumor, the number of cells identified for each CAFs subpopulation in each solid tumor type was documented in **Supplementary Table 4**. Compared with other CAFs subpopulations, apCAFs identified in the 9 solid tumor types display notable upregulation of diverse MHC class II molecules, such as CD74, HLA-DRA, and HLA-DRB1, resembling the apCAFs described by Elyada E et al. in PDAC (6) and Kerdidani D et al. in NSCLC (17). Moreover, by employing the graph-based clustering approach, we successfully segregated the cells within the T cells population and myeloid cells population of each solid tumor type into separate T cells subpopulations and myeloid cells subpopulations, respectively, using the classic markers specified in published articles (44–46) (**Supplementary Figures 6A-R**). Taken together, the main tumor, immune and stromal cell types, subpopulations of each main cell type, and especially apCAFs exist in these 9 tumors, indicating apCAFs’ potential important role by communicating with other different cell types.

### 3.2 apCAFs are associated with anti-tumor effects

**Figure 2:**
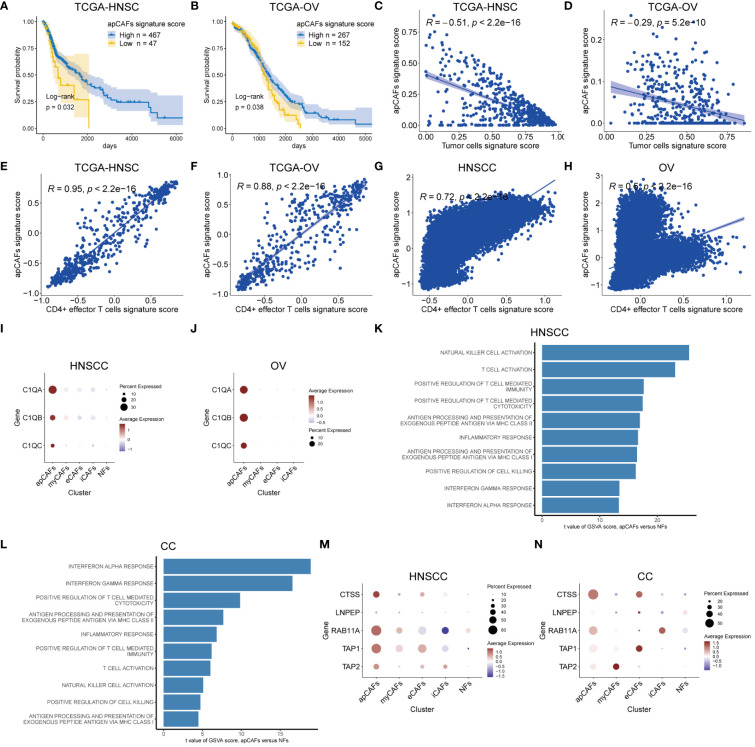
apCAFs are associated with anti-tumor effects. **(A, B)** Kaplan-Meier plots showing TCGA-HNSC cohort **(A)** and TCGA-OV cohort **(B)** patient survival probability by apCAFs signature scores. **(C, D)** Scatter plots showing Spearman’s correlation between the apCAFs signature scores and tumor cells signature scores in TCGA-HNSC cohort **(C)** and TCGA-OV cohort **(D)**. **(E, F)** Scatter plots showing Spearman’s correlation between the apCAFs gene signature scores and CD4+ effector T cells gene signature scores in TCGA-HNSC cohort **(E)** and TCGA-OV cohort **(F)**. **(G, H)** Scatter plots showing Spearman’s correlation between the apCAFs gene signature scores and CD4+ effector T cells gene signature scores in HNSCC **(G)** and OV **(H)**. **(I, J)** Dot plots showing the expression levels of C1Q molecules in distinct fibroblasts subpopulations of HNSCC **(I)** and OV **(J)**. **(K, L)** Bar plots showing the selected signaling pathways with significant enrichment of GO: BP and HALLMARK terms for apCAFs compared to NFs in HNSCC **(K)** and CC **(L)**. Differences in pathway activities scored per cell by GSVA between apCAFs and NFs. t values from a linear model, corrected for sample of origin. **(M, N)** Dot plots showing the expression profiles of molecule machinery involved in antigen processing and presentation in distinct fibroblasts subpopulations of HNSCC **(M)** and CC **(N)**. P-values were calculated by the log-rank test **(A, B)**. Dot size indicates fraction of expressing cells, colored based on normalized expression levels **(I, J, M, N)**. HNSCC, Head and Neck Squamous Cell Carcinoma; OV, Ovarian Cancer; CC, Cervical Cancer; apCAFs, antigen-presenting CAFs; myCAFs, myofibroblastic CAFs; eCAFs, extracellular matrix CAFs; iCAFs, inflammatory CAFs; NFs, normal fibroblasts.

Next, we wanted to know the relationship between apCAFs and tumor cells, since apCAFs were previously shown to have a pro-tumor function in PDAC (16) and an anti-tumor function in NSCLC (17). We obtained bulk RNA-seq cohorts including TCGA-HNSC (Head-Neck Squamous Cell Carcinoma), TCGA-OV (Ovarian Carcinoma), TCGA-CESC (Cervical Squamous Cell Carcinoma and Endocervical Adenocarcinoma), TCGA-COAD (Colon Adenocarcinoma), TCGA-READ (Rectum Adenocarcinoma), TCGA-BRCA (Breast Invasive Carcinoma), TCGA-SKCM (Skin Cutaneous Melanoma), and TCGA-KIRC (Kidney Renal Clear Cell Carcinoma) from the GDC database and GSE102349-NPC Nasopharyngeal Carcinoma RNA-seq cohort from the GEO database. By utilizing scRNA-seq data of the same solid tumor type with pre-annotated cell types as a reference dataset to make Signature Matrix (**Supplementary Table 5**), we applied the CIBERSORTx algorithm to perform deconvolution analysis on the bulk RNA-seq data of the same solid tumor type. As a result, we obtained signature scores for each sample in the bulk RNA-seq data, which represented the cells annotated by scRNA-seq (**Supplementary Table 6**). Utilizing clinical data obtained from the TCGA-HNSC cohort, we showed that higher signature scores of apCAFs were linked to improved prognosis outcome in HNSCC (**Figure 2A**). Moreover, we observed a robust inverse relationship between apCAFs signature scores and tumor cells signature scores in the TCGA-HNSC cohort, indicating a potential association of apCAFs with anti-tumor effects in HNSCC (**Figure 2C**). Similar analysis was performed in OV and we demonstrated that higher signature scores of apCAFs were linked to improved survival outcome and a noteworthy inverse relationship between apCAFs signature scores and tumor cells signature scores in the TCGA-OV cohort (**Figures 2B, D**). To generalize our findings, GSE102349-NPC, TCGA-CESC, TCGA-COAD, TCGA-READ, TCGA-BRCA, TCGA-SKCM, and TCGA-KIRC cohorts were used and we consistently showed that elevated signature scores of apCAFs were associated with improved survival outcome in most cancer types other than BRCA (**Supplementary Figures 7A-G**). Moreover, we consistently observed a strong negative correlation between apCAFs signature scores and tumor cells signature scores across the majority of solid tumor types, excluding RCC (**Supplementary Figures 7H-N**). Taken together, apCAFs are associated with anti-tumor effects in the majority of solid tumor types.

Considering the published report demonstrating the anti-tumor effects of apCAFs in NSCLC through direct activation of CD4+ effector T cells (17), we next examined the relationship between apCAFs and CD4+ effector T cells across various types of solid tumor. Seurat’s FindMarkers function was employed to identify the DEGs between apCAFs and other CAFs. We applied filters of logFC > 0.5 and adjPval < 0.05 to select significant DEGs. From these, we extracted the top 40 genes with the highest logFC as candidate genes for the apCAFs gene signature. Additionally, we obtained the gene signature for human CD4+ effector T cells from previously published articles (17) (**Supplementary Table 7**). For each solid tumor type, we used the apCAFs gene signature along with the CD4+ effector T cells gene signature as gene sets. Subsequently, we employed the GSVA R package to calculate the apCAFs gene signature scores and CD4+ effector T cells gene signature scores for each sample in the bulk RNA-seq data (**Supplementary Table 8**). We demonstrated a robust positive correlation between apCAFs gene signature scores and CD4+ effector T cells gene signature scores in the TCGA-HNSC (**Figure 2E**) and TCGA-OV (**Figure 2F**). Similarly, in 7 other cohorts like GSE102349-NPC, TCGA-CESC, TCGA-COAD, TCGA-READ, TCGA-BRCA, TCGA-SKCM, and TCGA-KIRC, we consistently showed a robust positive relationship between apCAFs gene signature scores and CD4+ effector T cells gene signature scores (**Supplementary Figures 7O-U**). Likewise, for every type of solid tumor, we employed the apCAFs gene signature in conjunction with the CD4+ effector T cells gene signature as gene sets. We calculated the apCAFs gene signature scores and CD4+ effector T cells gene signature scores for each cell within the scRNA-seq data of each solid tumor type, utilizing the AddModuleScore algorithm within the Seurat software. In both HNSCC (**Figure 2G**) and OV (**Figure 2H**), we have demonstrated a strong positive correlation between apCAFs gene signature scores and CD4+ effector T cells gene signature scores. Similarly, across 5 other scRNA-seq data cohorts including NPC, BRCA, AM, CM, and RCC, we consistently observed a robust positive association between apCAFs gene signature scores and CD4+ effector T cells gene signature scores (**Supplementary Figures 8A-E**). However, in CC scRNA-seq data cohort, there is a significant negative correlation between apCAFs gene signature scores and CD4+ effector T cells gene signature scores (**Supplementary Figure 8F**), while in CRC scRNA-seq data cohort, there is no significant correlation between apCAFs gene signature scores and CD4+ effector T cells gene signature scores (**Supplementary Figure 8G**).

In a previous study, it was shown that complement C1Q molecules expressed by apCAFs of NSCLC can directly bind to the corresponding receptors on CD4+ T cells, promoting the survival of CD4+ T cells (17). Therefore, we wondered whether apCAFs in other solid tumor types also express complement C1Q molecules. In the scRNA-seq data of HNSCC and OV, we observed a higher expression of complement C1Q molecules, namely C1QA, C1QB, and C1QC, in their apCAFs compared to other fibroblasts subpopulations (**Figures 2I, J**). Similarly, across the NPC, CC, CRC, BRCA, and AM scRNA-seq data cohorts, we consistently observed apCAFs exhibited elevated expression for complement C1Q molecules, specifically C1QA, C1QB, and C1QC, in contrast to other subpopulations of fibroblasts (**Supplementary Figures 9A-E**). Therefore, it appears that apCAFs may be associated with anti-tumor immune responses, possibly by promoting the survival of CD4+ T cells through C1Q molecules expression in these solid tumor types.

Intracellular pathways are generally responsible for the cell’s functionality. This led us to investigate the molecular pathways that were enriched in apCAFs within tumors compared to fibroblasts in normal tissues. The scRNA-seq datasets we collected contained normal tissue samples only from HNSCC, CC, NPC, and RCC solid tumor types. Therefore, we solely utilized the data from these solid tumor types to assess the alterations in signaling pathways of apCAFs. In brief, we conducted GSVA analysis to derive GSVA enrichment scores of the selected pathways for apCAFs and NFs in these solid tumor types. Subsequently, the limma R package was employed to analyze the differential expression of the selected pathways between apCAFs and NFs (**Supplementary Table 9**). This approach aimed to uncover the biological processes that contribute to the observed gene signature alterations. As a result, we observed that immune-related and inflammatory-related pathways were significantly enriched in apCAFs across these solid tumor types (**Figures 2K, L**; **Supplementary Figures 9F, G**). Surprisingly, when comparing apCAFs with NFs, we noted an enhanced capacity of apCAFs for promoting cell killing, activating NK cells and T cells, facilitating T cell-mediated immunity and cytotoxicity, as well as antigen presentation through MHC class I and MHC class II molecules. Moreover, we observed a consistent upregulation of antigen processing and presentation molecules, CTSS, LNPEP, RAB11A, TAP1, and TAP2, in apCAFs compared to other fibroblasts subpopulations in the majority of the solid tumor types based on the scRNA-seq data (**Figures 2M, N**; **Supplementary Figures 9H-N**). Taken together, these results strongly underscore the potential importance of apCAFs in the realm of anti-tumor immunity.

### 3.3 Illustration of the ST spots of various solid tumor tissues with apCAFs, tumor cells and CD4+ effector T cells signatures enrichment

**Figure 3:**
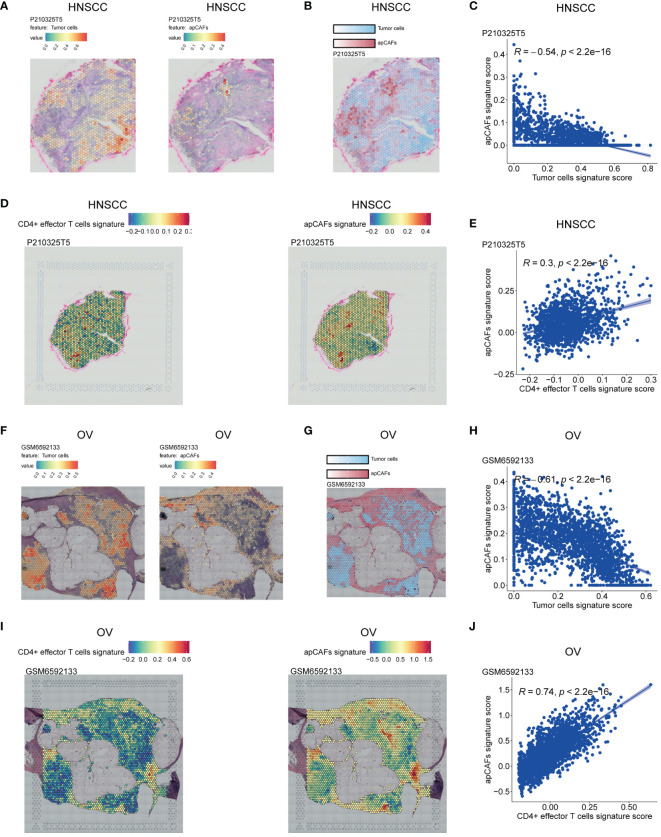
Illustration of the ST spots of various solid tumor tissues with apCAFs, tumor cells and CD4+ effector T cells signatures enrichment. **(A, F)** Left: Spatial transcriptomic spots with tumor cells signature enrichment in P210325T5 slice of HNSCC **(A)** and GSM6592133 slice of OV **(F)**; Right: Spatial transcriptomic spots with apCAFs signature enrichment in P210325T5 slice of HNSCC **(A)** and GSM6592133 slice of OV **(F)**. **(B, G)** Spatial transcriptomic spots with apCAFs and tumor cells signatures enrichment in one single plot in P210325T5 slice of HNSCC **(B)** and GSM6592133 slice of OV **(G)**. **(D, I)** Left: Spatial transcriptomic spots with CD4+ effector T cells gene signature enrichment in P210325T5 slice of HNSCC **(D)** and GSM6592133 slice of OV **(I)**; Right: Spatial transcriptomic spots with apCAFs gene signature enrichment in P210325T5 slice of HNSCC **(D)** and GSM6592133 slice of OV **(I)**. **(C, E, H, J)** Scatter plots showing Spearman’s correlation between apCAFs signature scores and both tumor cell signature scores and CD4+ effector T cell signature scores in the spatial transcriptomic spots in P210325T5 slice of HNSCC **(C, E)** and GSM6592133 slice of OV **(H, J)**. HNSCC, Head and Neck Squamous Cell Carcinoma; OV, Ovarian Cancer; apCAFs, antigen-presenting CAFs.

In order to examine the spatial relationship of apCAFs, tumor cells, and CD4+ effector T cells in greater detail, we leveraged the Visium ST data obtained from tumor tissue sections of patients with HNSCC, OV, BRCA, and CRC. In short, we employed semla R package to estimate cell type proportions from Visium ST spot expression profiles using an annotated scRNA-seq Seurat object of the same solid tumor type as a reference (**Supplementary Table 10**). Moreover, using the gene signatures of apCAFs originating from different solid tumor types and the gene signature of human CD4+ effector T cells, we employed the Seurat function AddModuleScore to determine gene signature scores for both the apCAFs gene signature and the CD4+ effector T cell gene signature at each spatial spot on the tissue slices (**Supplementary Table 11**). In the HNSCC slice, we observed distinct separation in the distribution of enriched regions for tumor cells and apCAFs signatures. The areas enriched with tumor cells signature exhibited low apCAFs signature, while the regions enriched with apCAFs signature showed reduced tumor cells signature (**Figures 3A, B**; **Supplementary Figures 10A, B, F, G**). Furthermore, a consistent inverse correlation between tumor cells signature scores and apCAFs signature scores was apparent in the ST spots of HNSCC (**Figure 3C**; **Supplementary Figures 10C, H**). Moreover, in the HNSCC slice, we observed a co-localization distribution pattern of enriched regions for CD4+ effector T cells gene signature and apCAFs gene signature. In other words, the regions enriched with CD4+ effector T cells gene signature exhibited elevated apCAFs gene signature and the areas enriched with apCAFs gene signature showed increased CD4+ effector T cells gene signature (**Figure 3D**; **Supplementary Figures 10D, I**). In addition, within the ST spots of HNSCC, a pronounced positive association between CD4+ effector T cells gene signature scores and apCAFs gene signature scores was observed (**Figure 3E**; **Supplementary Figures 10E, J**). Similarly, we noted clear separation in the distribution of enriched regions for tumor cells and apCAFs signatures in slices of OV (**Figures 3F, G**; **Supplementary Figures 11A, B, F, G**), BRCA (**Supplementary Figures 12A, B, F, G, K, L**), and CRC (**Supplementary Figures 13A, B, F, G**). Moreover, a steady negative relationship between the signature scores of tumor cells and apCAFs was consistently visible in the ST spots of OV (**Figure 3H**; **Supplementary Figures 11C, H**), BRCA (**Supplementary Figures 12C, H, M**), and CRC (**Supplementary Figures 13C, H**). Furthermore, in the slices of OV (**Figure 3I**; **Supplementary Figures 11D, I**), BRCA (**Supplementary Figures 12D, I, N**), and CRC (**Supplementary Figures 13D, I**), a co-localization in the distribution of enriched regions for CD4+ effector T cells gene signature and apCAFs gene signature was noted. In addition, within the ST spots of OV (**Figure 3J**; **Supplementary Figures 11E, J**), BRCA (**Supplementary Figures 12E, J, O**), and CRC (**Supplementary Figures 13E, J**), a significant positive relationship between the gene signature scores of CD4+ effector T cells and apCAFs was evident.

Collectively, the spatially inverse relationship between tumor cells and apCAFs across these 4 solid tumor types suggests that apCAFs are associated with tumor suppression. Moreover, across these 4 types of solid tumor, CD4+ effector T cells and apCAFs exhibit a co-localized spatial distribution, indicating that apCAFs may be associated with anti-tumor effects by promoting the survival of CD4+ T cells.

### 3.4 The correlation between apCAFs and T cells subpopulations

**Figure 4:**
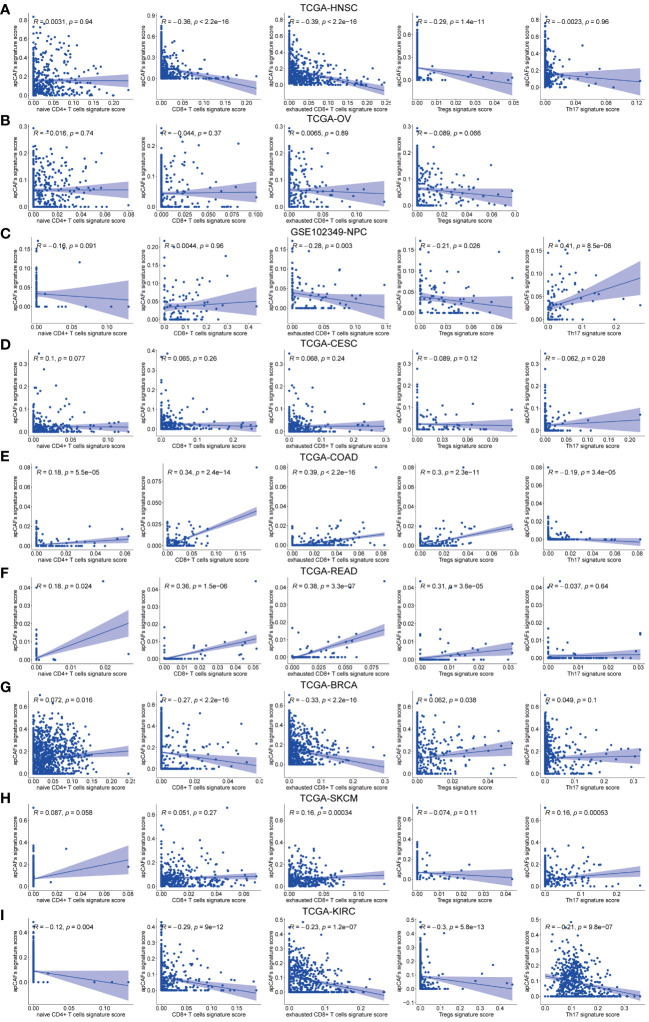
The correlation between apCAFs and T cells subpopulations. **(A-I)** Scatter plots showing Spearman’s correlation between the apCAFs and naïve CD4+ T cells, total CD8+ T cells, exhausted CD8+ T cells, Tregs, Th17 cells signature scores in TCGA-HNSC cohort **(A)**, TCGA-OV cohort **(B)**, GSE102349-NPC cohort **(C)**, TCGA-CESC cohort **(D)**, TCGA-COAD cohort **(E)**, TCGA-READ cohort **(F)**, TCGA-BRCA cohort **(G)**, TCGA-SKCM cohort **(H)**, and TCGA-KIRC cohort **(I)**. NPC, Nasopharyngeal Carcinoma; apCAFs, antigen-presenting CAFs.

Given that different T cells subpopulations normally co-exist in the TME, we performed correlation studies between apCAFs and other T cells subpopulations, e.g., naïve CD4+ T cells, total CD8+ T cells, exhausted CD8+ T cells, Tregs, Th17 cells. We first utilized scRNA-seq data of the same solid tumor type with pre-annotated apCAFs and T cells subpopulations as a reference dataset to generate a signature matrix. Then, we applied the CIBERSORTx algorithm to perform deconvolution analysis on the bulk RNA-seq data of the same solid tumor type. Thus, we obtained cell type signature scores for each sample in the bulk RNA-seq data. In the TCGA-HNSC cohort, apCAFs were significantly negatively correlated with total CD8+ T cells, exhausted CD8+ T cells, and Tregs (**Figure 4A**). In the TCGA-OV and TCGA-CESC cohorts, there was no correlation between apCAFs and all types of T cells subpopulations (**Figures 4B, D**). In the GSE102349-NPC cohort, apCAFs were significantly negatively correlated with exhausted CD8+ T cells and Tregs, while significantly positively correlated with Th17 cells (**Figure 4C**). In the TCGA-COAD and TCGA-READ cohorts, apCAFs were significantly positively correlated with naïve CD4+ T cells, total CD8+ T cells, exhausted CD8+ T cells, and Tregs (**Figures 4E, F**). In the TCGA-BRCA cohort, apCAFs were significantly negatively correlated with total CD8+ T cells and exhausted CD8+ T cells (**Figure 4G**). In the TCGA-SKCM cohort, apCAFs showed a weak positive correlation with exhausted CD8+ T cells and Th17 cells (**Figure 4H**). In the TCGA-KIRC cohort, apCAFs were negatively correlated with all types of T cells subpopulations (**Figure 4I**). Taken together, apCAFs could be negatively or positively correlated with other T cells subpopulations or even no correlation, mostly depending on the tumor types, suggesting apCAFs probably involved in regulating T cells mediated immune response despite their heterogeneity.

### 3.5 Characterization of apCAFs origin

**Figure 5:**
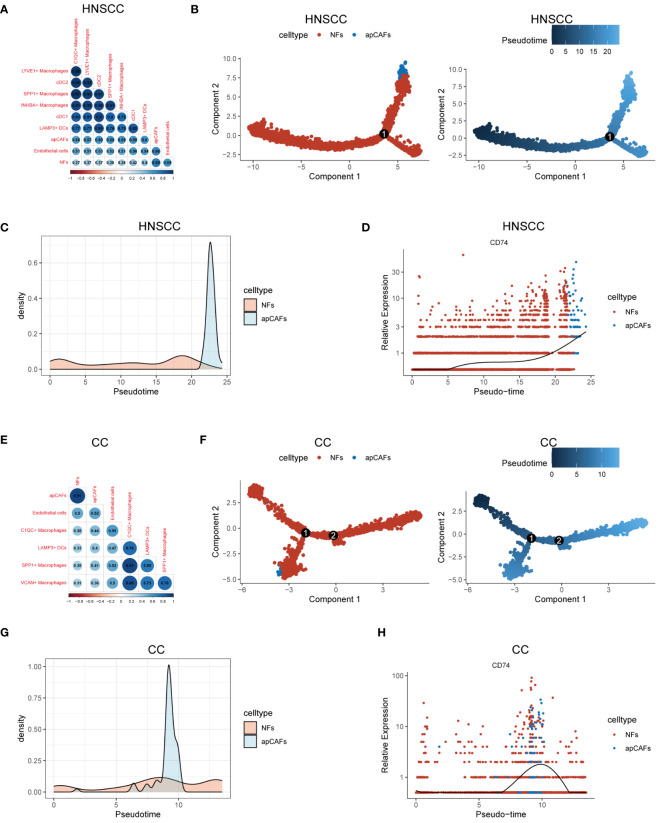
Characterization of apCAFs origin. **(A, E)** Transcriptomic similarity analyses among apCAFs, NFs, endothelial cells, various macrophages and various dendritic cells in HNSCC **(A)** and CC **(E)**. The darker the blue, the stronger the positive correlation, and the darker the red, the stronger the negative correlation. The numbers in the circle represent the correlation coefficient R. **(B, F)** Left: The cell trajectory along the NFs-apCAFs path in HNSCC **(B)** and CC **(F)**; Right: The pseudotime trajectory along the NFs-apCAFs path in HNSCC **(B)** and CC **(F)**. **(C, G)** Density distribution of apCAFs and NFs along the pseudotime trajectory in HNSCC **(C)** and CC **(G)**. **(D, H)** Dynamic variation in CD74 during pseudotime trajectory in HNSCC **(D)** and CC **(H)**. HNSCC, Head and Neck Squamous Cell Carcinoma; CC, Cervical Cancer; apCAFs, antigen-presenting CAFs; NFs, normal fibroblasts; cDC1, conventional type 1 dendritic cells; cDC2, conventional type 2 dendritic cells; LAMP3+ DCs, LAMP3+ dendritic cells.

Previous studies have shown that macrophages (47, 48), DCs (15), and endothelial cells (48, 49) can undergo a transformation into fibroblasts under certain conditions, suggesting that these cells could be potential sources of apCAFs. To investigate the potential cellular origins of apCAFs, we conducted transcriptomics similarity analysis between apCAFs and their potential source cell types (**Supplementary Table 12**). In HNSCC, we observed a strong transcriptional resemblance between apCAFs and NFs (R = 0.69) (**Figure 5A**). This finding suggests that NFs are likely the primary source of apCAFs in HNSCC. Notably, pseudotime trajectory analysis using Monocle R package provided insights into a potential differentiation path from NFs to apCAFs (**Figures 5B, C**). Within the pseudotime trajectory of NFs differentiation into apCAFs in HNSCC, we observed an upregulation of CD74, a signature gene of apCAFs, along the trajectory (**Figure 5D**). Similarly, our analysis revealed that apCAFs displayed the highest transcriptional similarity with NFs in CC (**Figure 5E**), NPC (**Supplementary Figure 14A**), and RCC (**Supplementary Figure 14E**), with R of 0.91, 0.85, and 0.57, respectively. Through pseudotime trajectory analysis, we observed a potential evolutionary path from NFs to apCAFs in CC (**Figures 5F, G**), NPC (**Supplementary Figures 14B, C**), and RCC (**Supplementary Figures 14F, G**). Remarkably, during pseudotime trajectory, the expression level of CD74, a signature gene of apCAFs, was consistently upregulated in CC (**Figure 5H**), NPC (**Supplementary Figure 14D**), and RCC (**Supplementary Figure 14H**). Our data strongly suggests that NFs are the likely origin of apCAFs in these solid tumor types.

### 3.6 Transcription factors enrichment in apCAFs

**Figure 6:**
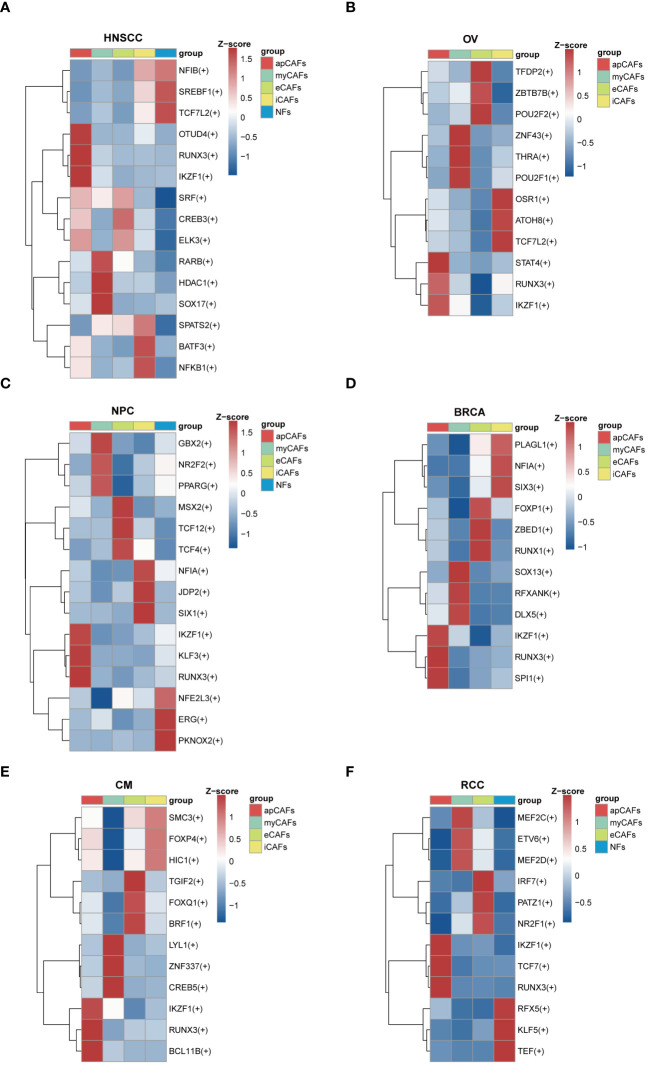
Transcription factors enrichment in apCAFs. **(A-F)** Top 3 regulons enrichment of NFs and various CAFs via SCENIC analysis in HNSCC **(A)**, OV **(B)**, NPC **(C)**, BRCA **(D)**, CM **(E)**, RCC **(F)**. Square colors represent different fibroblasts subpopulations. Scale bar represents Z-score. Symbol “(+)” indicates positive regulation by the transcription factor. HNSCC, Head and Neck Squamous Cell Carcinoma; OV, Ovarian Cancer; NPC, Nasopharyngeal Carcinoma; BRCA, Breast Cancer; CM, Cutaneous Melanoma; RCC, Renal Cell Carcinoma; apCAFs, antigen-presenting CAFs; myCAFs, myofibroblastic CAFs; eCAFs, extracellular matrix CAFs; iCAFs, inflammatory CAFs; NFs, normal fibroblasts.

Since transcription factors (TFs) are essential for cell differentiation, we applied the SCENIC software to identify highly activated TFs within apCAFs (**Supplementary Table 13**). Utilizing the pySCENIC python package, we conducted a comprehensive analysis to pinpoint TFs in apCAFs that could contribute to their functional attributes. In our findings, we noted the enrichment of TFs such as OTUD4, RUNX3, and IKZF1 in HNSCC (**Figure 6A**), STAT4, RUNX3, and IKZF1 in OV (**Figure 6B**), IKZF1, KLF3, and RUNX3 in NPC (**Figure 6C**), IKZF1, RUNX3, and SPI1 in BRCA (**Figure 6D**), IKZF1, RUNX3, and BCL11B in CM (**Figure 6E**), and IKZF1, TCF7, and RUNX3 in RCC (**Figure 6F**). Taken together, our analysis revealed that RUNX3 and IKZF1 were consistently enriched in apCAFs across 6 solid tumor types, indicating that they may be associated with the formation of apCAFs.

### 3.7 apCAFs exhibit a distinct glycolytic metabolic pattern

**Figure 7:**
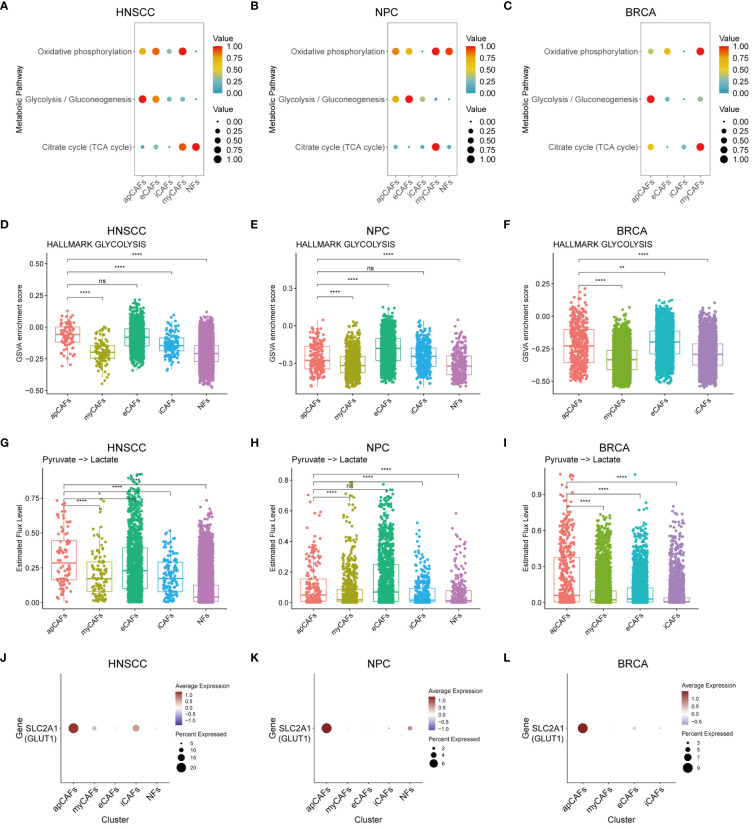
apCAFs exhibit a distinct glycolytic metabolic pattern. **(A-C)** Metabolic pathway enriched in different fibroblasts subpopulations within HNSCC **(A)**, NPC **(B)**, and BRCA **(C)** through the utilization of scMetabolism algorithm. **(D-F)** Boxplots showing glycolysis pathway of HALLMARK term enriched in distinct fibroblasts subpopulations within HNSCC **(D)**, NPC **(E)**, and BRCA **(F)** using GSVA algorithm. **(G-I)** Boxplots showing relative level of the estimated metabolic flux from pyruvate to lactate in distinct fibroblasts subpopulations of HNSCC **(G)**, NPC **(H)**, and BRCA **(I)** using scFEA algorithm. **(J-L)** Dot plots showing the expression profiles of glucose transporter 1 (GLUT1) gene SLC2A1 in distinct fibroblasts subpopulations of HNSCC **(J)**, NPC **(K)**, and BRCA **(L)**. ns, *, **, and **** represent P-value > 0.05, P-value ≤ 0.05, P-value ≤ 0.01, and P-value ≤ 0.0001, respectively, P-value was calculated from Wilcoxon rank sum test **(D-I)**. HNSCC, Head and Neck Squamous Cell Carcinoma; NPC, Nasopharyngeal Carcinoma; BRCA, Breast Cancer; apCAFs, antigen-presenting CAFs; myCAFs, myofibroblastic CAFs; eCAFs, extracellular matrix CAFs; iCAFs, inflammatory CAFs; NFs, normal fibroblasts.

As is known, metabolic processes regulate the function of immune cells (50). We applied the scMetabolism algorithm (37) to analyze scRNA-seq data of fibroblasts subpopulations in various solid tumor types, and our analysis revealed a significant increase in the enrichment of the glycolysis and gluconeogenesis pathway in apCAFs as compared to other fibroblasts subpopulations within solid tumor types, including HNSCC (**Figure 7A**), NPC (**Figure 7B**), and BRCA (**Figure 7C**). To further validate the enrichment of the glycolytic pathway in apCAFs, we employed the GSVA algorithm to calculate the GSVA enrichment scores of the glycolysis pathway of HALLMARK term in fibroblasts subpopulations from various solid tumor types (**Supplementary Table 14**). We observed that the glycolysis pathway GSVA enrichment scores of apCAFs were higher compared with all other fibroblasts subpopulations except for eCAFs in solid tumor types including HNSCC (**Figure 7D**), NPC (**Figure 7E**), and BRCA (**Figure 7F**). Moreover, using the single-cell metabolic flux estimation algorithm scFEA, we discovered that the metabolic flux from pyruvate to lactate in apCAFs was higher compared to other fibroblasts subpopulations in HNSCC (**Figure 7G**), NPC (**Figure 7H**), and BRCA (**Figure 7I**) (**Supplementary Table 15**). Furthermore, glucose transporter 1 (GLUT1) serves as a pivotal regulatory protein in glycolysis, aiding in the translocation of glucose across the plasma membranes of most cells of the body (51). Interestingly, we found that apCAFs exhibited higher expression of the GLUT1 gene, SLC2A1, compared with other fibroblasts subpopulations in solid tumor types including HNSCC (**Figure 7J**), NPC (**Figure 7K**), and BRCA (**Figure 7L**). Collectively, all these lines of evidence strongly support that in certain solid tumors, apCAFs may be associated with a notable glycolytic metabolic pattern, consistent with the notion of metabolism regulating immunity.

## 4 Discussion

Our study is the first to perform a comprehensive pan-cancer analysis of apCAFs in 9 solid tumor types despite several large bioinformatical analyses reporting heterogeneity, plasticity (48) and prognostic value of CAFs (52). We first have shown that apCAFs are not limited to a few specific types of solid tumor but rather generally exist within the microenvironment of various solid tumor types, which largely extends the findings of apCAFs presence in pancreatic cancer (6) and breast cancer (53). Then, our analysis revealed that a higher apCAFs signature scores correlated with improved survival outcomes in HNSCC, OV, NPC, CC, CRC, CM, and RCC. Additionally, a noteworthy inverse relationship between the signature scores of apCAFs and tumor cells was observed in HNSCC, OV, NPC, CC, CRC, BRCA, and CM. Moreover, we observed a spatial inverse correlation between tumor cells and apCAFs within spatial data from 4 out of the 9 solid tumor types by spatial transcriptomics analysis. These results suggest that apCAFs may be associated with anti-tumor effects in these solid tumor types, consistent with the anti-tumor effect observed in NSCLC (17), but contrasting with the tumor-promoting effect found in PDAC (16). However, it is essential to conduct further studies to comprehensively elucidate the precise mechanism involved.

The findings by Huang H et al. revealed that apCAFs were capable of selectively driving the differentiation of naive CD4+ T cells into Tregs in response to antigens in PDAC (16). Furthermore, Elyada E et al. have proposed a theory proposing that apCAFs use MHC class II molecules as decoy receptors to render CD4+ T cells nonfunctional, either through the induction of anergy or by facilitating their differentiation into Tregs, particularly in the context of PDAC (6). Conversely, Kerdidani D et al. reported that apCAFs directly activated CD4+ effector T cells in NSCLC and promoted the survival of CD4+ effector T cells through the expression of C1Q molecules and proposed an innovative conceptual framework suggesting that efficient MHC class II immunity in NSCLC requires *in situ* antigen presentation by CD4+ T cells within the TME (17). Extending these findings, our results revealed a notable positive relationship between the apCAFs gene signature and the CD4+ effector T cells gene signature across HNSCC, OV, NPC, CC, CRC, BRCA, CM, and RCC and apCAFs displayed increased expression levels of complement C1Q molecules. In addition, we identified spatial co-localization between CD4+ effector T cells and apCAFs in 4 solid tumor types. We thus reason that apCAFs may potentially be associated with anti-tumor immune effects by promoting the survival of CD4+ effector T cells through the reported mechanism of expressing C1Q molecules. Additionally, our analysis results indicate a significant negative correlation between apCAFs and exhausted CD8+ T cells in HNSCC, NPC, BRCA, and RCC, suggesting a potential negative association between apCAFs and immunosuppression in these cancers. In HNSCC, NPC, and RCC, apCAFs show a significant negative correlation with Tregs, similar to findings where CAFs expressing the macrophage classical marker CD68 in oral squamous cell carcinoma is found to inhibit Tregs infiltration (54). Moreover, in NPC, CM, and RCC, apCAFs exhibit significant correlations with Th17 cells, while Th17 cells have dual roles in promoting and inhibiting tumor development (55, 56). In CRC and CM, apCAFs are significantly positively correlated with exhausted CD8+ T cells, akin to findings in CRC where CAFs driven antigen cross-presentation via MHC class I molecules led to CD8+ T cells exhaustion (57), and in a B16 cell model where CAFs mediated antigen cross-presentation via MHC class I molecules weaken CD8+ T cells cytotoxic effects (58). In CRC, apCAFs are significantly positively correlated with Tregs, similar to findings where IL1R2 expressed by Tregs in a mouse MC38 cell tumor model enhances the interaction between Tregs and CAFs by upregulating MHC class II molecules on CAFs (59). All of these suggest that apCAFs are linked with different T cells mediated immune response and the microenvironment difference in different solid tumor types may result in varying relationships between apCAFs and T cells subpopulations. The functional role of apCAFs on different T cells and underlying mechanism are warranted to be investigated in the future.

Compared with NFs, intrinsic signaling pathway enrichment analysis showed apCAFs possessed an enhanced ability to promote cell killing, activate NK cells, induce T cell activation, strengthen T-cell-mediated immunity, facilitate T-cell-mediated cytotoxicity, and enable antigen presentation via MHC class I and MHC class II. Moreover, across the majority of the solid tumor types we collected, antigen processing and presentation molecules expression increased in apCAFs. Furthermore, apCAFs showed greater responsiveness to interferon and inflammatory signals in comparison to NFs. These results align with earlier studies indicating that the IFN-γ induce MHC class II molecules expression in fibroblasts (60). This suggests that interferon has the potential to enhance apCAFs, leading to the activation of CD4+ effector T cells within tumors and provides valuable insights for the future development of treatments for solid tumor. All these findings provide support for the “2nd hit hypothesis” proposed by Tsoumakidou M, which suggests that complete T cells activation requires *in situ* antigen presentation by apCAFs, besides interactions with professional APCs such as DCs, macrophages, and B cells (18). Consequently, apCAFs may be associated with anti-tumor effects through these mechanisms. However, there is a need for further studies to fully elucidate the precise mechanisms involved. Additionally, we found that apCAFs subpopulation identified in most solid tumor types exhibit higher expression of immune checkpoint receptors such as TIGIT, LAG3, and CTLA4 compared to other fibroblast subpopulations (**Supplementary Figures 8H-P**). These immune checkpoint receptors are primarily expressed on cells such as CD8+ T cells and CD4+ T cells, which usually inhibit activation and function of these cells (61–63). The functional role of these immune checkpoint receptors in apCAFs is warranted to be investigated in the future.

Our data indicate that NFs are likely to be the primary source of apCAFs in HNSCC, CC, NPC, and RCC. These results are consistent with the earlier study conducted by Sebastian A et al., where they identified cells displaying the apCAFs signature in both normal breast tissue and breast cancer (64). This consistency strengthens the reliability of our results. Moreover, Luo H et al. speculated that apCAFs might represent a transitional state between myCAFs and tumor-associated macrophages (TAMs) (48). Furthermore, Kerdidani D et al. have provided evidence that apCAFs originated from alveolar epithelial cells in lung cancer (17), and Huang H et al. reported that apCAFs have been revealed to have their origin in mesothelial cells within pancreatic cancer (16). TAMs, alveolar epithelial cells, and mesothelial cells also serve as potential sources of apCAFs. Moreover, in PDAC, apCAFs exhibited an enrichment of nuclear factor κB (NF-κB) signaling and transforming growth factor β (TGF-β) signaling compared to their source cells, mesothelial cells (16). This is distinct from apCAFs in solid tumor types such as HNSCC, CC, NPC, and RCC, where an enrichment in immune response pathways, such as T cells activation and NK cells activation, relative to NFs, was observed. Although both apCAFs in PDAC and apCAFs in certain solid tumor types such as HNSCC, CC, NPC, and RCC express MHC class II molecules like CD74, the potential transformation paths and enriched signaling pathways of these 2 apCAFs types greatly differ, possibly accounting for the pro-tumor effects of apCAFs in PDAC and the association of apCAFs with anti-tumor effects in certain solid tumor types like HNSCC, CC, NPC, and RCC. However, future experiments utilizing labeling and lineage tracing methods will be useful to validate the origin of apCAFs.

In addition, our findings revealed that the TFs RUNX3 and IKZF1 were significantly enriched in apCAFs across 6 solid tumor types. This is consistent with previous findings in which apCAFs of hepatocellular carcinoma were found to be enriched in TFs RUNX3 and IKZF1 (65). Study have shown that RUNX3 was necessary for the maturation of splenic DCs, whereas RUNX3-deficient splenic DCs displayed impaired expression of MHC class II molecules and diminished T cells priming activity (66). Therefore, the enrichment of the RUNX3 in apCAFs may be associated with the MHC class II molecules expression and T cell priming of apCAFs. Moreover, IKZF1 plays essential roles throughout various phases of lymphocyte development and hematopoiesis (67). Hence, the enrichment of the IKZF1 in apCAFs may be associated with the formation or function of apCAFs. RUNX3 and IKZF1 could potentially be involved in either the formation or functionality of apCAFs. However, the precise underlying molecular mechanism by which RUNX3 and IKZF1 promote apCAFs formation requires additional investigation.

The functions of immune cells and host immunity are affected by their metabolic processes (68). For instance, effector T cells primarily exhibit a glycolytic phenotype (69), whereas the anti-inflammatory Tregs rely on mitochondrial oxidative phosphorylation (70). In addition, metabolic patterns, especially glycolysis, also affect the differentiation, activation, and antigen-presenting function of APCs (71, 72). Particularly, a recent article has reported that fibroblastic reticular cells in B cell lymphoma exhibited enhanced antigen presentation and glycolysis pathways relative to normal tissue’s fibroblastic reticular cells (73). Similarly, our analysis revealed that apCAFs originating from solid tumor types such as HNSCC, NPC, and BRCA are associated with enriched glycolytic metabolic patterns, with higher expression of the glucose transporter GLUT1. These results propose the idea that glycolysis could be related to the functions of apCAFs in HNSCC, NPC, and BRCA, potentially analogous to the role of glycolysis in DCs. For example, studies have shown that monocyte-derived DCs required glycolysis to support the expression of their MHC class II molecules (74), while the inhibition of glycolysis in DCs within the local draining lymph nodes of mice could lead to decreased antigen presentation capacity of DCs and subsequent decreased percentages of activated antigen specific Th17 cells (75).

## 5 Conclusions

In conclusion, our findings showcase that apCAFs, likely primarily derive from NFs, are prevalent in various solid tumors and generally are associated with anti-tumor effects. apCAFs may be linked to the activation of CD4+ effector T cells and potentially promote the survival of CD4+ effector T cells through the expression of C1Q molecules. Moreover, apCAFs exhibit specific enrichment of TFs RUNX3 and IKZF1, along with significant glycolytic metabolism. All these results provide novel insights into a deeper understanding of apCAFs and the potential therapeutic implications of apCAFs-targeted cancer immunotherapy.

## Data availability statement

Publicly available datasets were analyzed in this study. Expression data that comes after can be accessed from a range of repositories, including the Gene Expression Omnibus (GEO), the Genome Sequence Archive for Human (GSA for Human), the European Genome-Phenome Archive (EGA), the Zenodo data repository, and the Genomic Data Commons (GDC) database. The specific studies included in the analyses are provided in **Supplementary Tables 1**. Previously published scRNA-seq data analyzed in this study can be accessed through the following accession codes: GSE181919 [Head and neck cancer data by Choi JH et al. available from the GEO (76)], GSE165897 [Ovarian cancer data by Zhang K et al. available from the GEO (77)], GSE208653 [Cervical cancer data by Guo C et al. available from the GEO (78)], GSE176078 [Breast cancer data by Wu SZ et al. available from the GEO (79)], GSE189889 [Acral melanoma data by Li J et al. available from the GEO (80)], GSE215120 [Cutaneous melanoma data by Zhang C et al available from the GEO (81)], HRA000087 [Nasopharyngeal carcinoma data by Jin S et al. available from the GSA for Human (46)], HRA000979 [Colorectal cancer data by Qi J et al. available from the GSA for Human (44)], and EGAD00001008030 [Renal cell carcinoma data by Li R et al. available from the EGA (82)]. Previously published ST data reanalyzed in this study can be accessed through the following accession codes: GSE181300 [Head and neck cancer data by Cheng HY et al. available from the GEO (83)], GSE213699 [Ovarian cancer data by Sanders BE et al. available from the GEO (84)], GSE226997 [Colorectal cancer data by Park SS et al. available from the GEO (85)], and DOI 10.5281/zenodo.4739739 [Breast cancer data by Wu SZ et al. available from the Zenodo data repository (79)]. Previously published bulk RNA-seq data reanalyzed in this study can be accessed through the following accession codes: GSE102349 [Nasopharyngeal carcinoma data by Zhang L et al. available from the GEO (86)], and TCGA-HNSC, TCGA-SKCM, TCGA-BRCA, TCGA-COAD, TCGA-READ, TCGA-KIRC, TCGA-OV, and TCGA-CESC from the GDC database.

## Author contributions

JC: Conceptualization, Formal analysis, Writing – original draft. RC: Formal analysis, Writing – original draft. JH: Conceptualization, Supervision, Writing – review & editing.

## Supplementary material

Supplementary Figure 1 Quality control of overall single-cell transcriptome profiles. **(A-I)** Violin plots of nFeature_RNA, nCount_RNA and percent.mt of all samples in HNSCC **(A)**, CM **(B)**, OV **(C)**, CC **(D)**, NPC **(E)**, CRC **(F)**, BRCA **(G)**, AM **(H)** and RCC **(I)**. The identities under the horizontal axis represent the source of the sample. HNSCC, Head and Neck Squamous Cell Carcinoma; CM, Cutaneous Melanoma; OV, Ovarian Cancer; CC, Cervical Cancer; NPC, Nasopharyngeal Carcinoma; CRC, Colorectal Cancer; BRCA, Breast Cancer; AM, Acral Melanoma; RCC, Renal Cell Carcinoma.

Supplementary Figure 2 Batch effects of overall single-cell transcriptome profiles. **(A-I)** UMAP plots of the overall 43507 cells in HNSCC **(A)**, 46116 cells in OV **(B)**, 236707 cells in NPC **(C)**, 58964 cells in CC **(D)**, 37345 cells in CRC **(E)**, 77196 cells in BCRA **(F)**, 181677 cells in AM **(G)**, 23452 cells in CM **(H)** and 176664 cells in RCC **(I)**, with each cell color coded for the sample of origin. HNSCC, Head and Neck Squamous Cell Carcinoma; OV, Ovarian Cancer; NPC, Nasopharyngeal Carcinoma; CC, Cervical Cancer; CRC, Colorectal Cancer; BRCA, Breast Cancer; AM, Acral Melanoma; CM, Cutaneous Melanoma; RCC, Renal Cell Carcinoma.

Supplementary Figure 3 Hierarchical clustering of overall single-cell transcriptome profiles. **(A-I)** Clustering trees showing hierarchical clustering for HNSCC **(A)**, OV **(B)**, NPC **(C)**, CC **(D)**, CRC **(E)**, BRCA **(F)**, AM **(G)**, CM **(H)** and RCC **(I)**. HNSCC, Head and Neck Squamous Cell Carcinoma; OV, Ovarian Cancer; NPC, Nasopharyngeal Carcinoma; CC, Cervical Cancer; CRC, Colorectal Cancer; BRCA, Breast Cancer; AM, Acral Melanoma; CM, Cutaneous Melanoma; RCC, Renal Cell Carcinoma.

Supplementary Figure 4 Hierarchical clustering of overall CAFs single-cell transcriptome profiles. **(A-I)** Clustering trees showing hierarchical clustering for CAFs in HNSCC **(A)**, OV **(B)**, NPC **(C)**, CC **(D)**, CRC **(E)**, BRCA **(F)**, AM **(G)**, CM **(H)** and RCC **(I)**. HNSCC, Head and Neck Squamous Cell Carcinoma; OV, Ovarian Cancer; NPC, Nasopharyngeal Carcinoma; CC, Cervical Cancer; CRC, Colorectal Cancer; BRCA, Breast Cancer; AM, Acral Melanoma; CM, Cutaneous Melanoma; RCC, Renal Cell Carcinoma.

Supplementary Figure 5 Identification of apCAFs in various solid tumor types. **(A, C, E, G, I, K, M)** Left: UMAP plots showing the major cell types in NPC **(A)**, CC **(C)**, CRC **(E)**, BRCA **(G)**, AM **(I)**, CM **(K)** and RCC **(M)**; Right: Dot plots showing selected cell marker genes expression levels of the major cell types in NPC **(A)**, CC **(C)**, CRC **(E)**, BRCA **(G)**, AM **(I)**, CM **(K)** and RCC **(M)**. **(B, D, F, H, J, L, N)** Left: UMAP plots showing 4 major subpopulations of CAFs in NPC **(B)**, CC **(D)**, CRC **(F)**, BRCA **(H)**, 5 major subpopulations of CAFs in AM **(J)**, 4 major subpopulations of CAFs in CM **(L)** and 3 major subpopulations of CAFs in RCC **(N)**. Right: Dot plots showing selected cell marker genes expression levels for each subpopulation of CAFs in NPC **(B)**, CC **(D)**, CRC **(F)**, BRCA **(H)**, AM **(J)**, CM **(L)** and RCC **(N)**. Dot size indicates fraction of expressing cells, colored based on normalized expression levels **(A-N:** Right**)**. NPC, Nasopharyngeal Carcinoma; CC, Cervical Cancer; CRC, Colorectal Cancer; BRCA, Breast Cancer; AM, Acral Melanoma; CM, Cutaneous Melanoma; RCC, Renal Cell Carcinoma; apCAFs, antigen-presenting CAFs; myCAFs, myofibroblastic CAFs; eCAFs, extracellular matrix CAFs; iCAFs, inflammatory CAFs, pDCs, plasmacytoid dendritic cells.

Supplementary Figure 6 Myeloid cells and T cells subpopulations in various solid tumor types. **(A, C, E, G, I, K, M, O, Q)** Left: UMAP plots showing 7 major subpopulations of myeloid cells in HNSCC **(A)**, 6 major subpopulations of myeloid cells in OV **(C)**, NPC **(E)**, CC **(G)**, 5 major subpopulations of myeloid cells in CRC **(I)**, 6 major subpopulations of myeloid cells in BRCA **(K)**, 5 major subpopulations of myeloid cells in AM **(M)**, CM **(O)**, and 6 major subpopulations of myeloid cells in RCC **(Q)**. Right: Dot plots showing selected cell marker genes expression levels for the major subpopulations of myeloid cells in HNSCC **(A)**, OV **(C)**, NPC **(E)**, CC **(G)**, CRC **(I)**, BRCA **(K)**, AM **(M)**, CM **(O)** and RCC **(Q)**. **(B, D, F, H, J, L, N, P, R)** Left: UMAP plots showing 3 major subpopulations of T cells in HNSCC **(B)**, OV **(D)**, 4 major subpopulations of T cells in NPC **(F)**, 3 major subpopulations of T cells in CC **(H)**, 4 major subpopulations of T cells in CRC **(J)**, BRCA **(L)**, and 3 major subpopulations of T cells in AM **(N)**, CM **(P)**, RCC **(R)**. Right: Dot plots showing selected cell marker genes expression levels for the major subpopulations of T cells in HNSCC **(B)**, OV **(D)**, NPC **(F)**, CC **(H)**, CRC **(J)**, BRCA **(L)**, AM **(N)**, CM **(P)** and RCC **(R)**. Dot size indicates fraction of expressing cells, colored based on normalized expression levels **(A-R:** Right**)**. HNSCC, Head and Neck Squamous Cell Carcinoma; OV, Ovarian Cancer; NPC, Nasopharyngeal Carcinoma; CC, Cervical Cancer; CRC, Colorectal Cancer; BRCA, Breast Cancer; AM, Acral Melanoma; CM, Cutaneous Melanoma; RCC, Renal Cell Carcinoma; cDC1, conventional type 1 dendritic cells; cDC2, conventional type 2 dendritic cells; LAMP3+ DCs, LAMP3+ dendritic cells; pDCs, plasmacytoid dendritic cells; Tregs, regulatory T cells.

Supplementary Figure 7 apCAFs show the anti-tumor effects. **(A)** Kaplan-Meier plot showing progression free survival rate of patients by apCAFs signature scores in GSE102349-NPC RNA-seq cohort. **(B-G)** Kaplan-Meier plots showing overall survival probability of patients by apCAFs signature scores in TCGA-CESC cohort **(B)**, TCGA-COAD cohort **(C)**, TCGA-READ cohort **(D)**, TCGA-BRCA cohort **(E)**, TCGA-SKCM cohort **(F)** and TCGA-KIRC cohort **(G)**. (**H**-**N)** Scatter plots showing Spearman’s correlation between the apCAFs signature scores and tumor cells signature scores in GSE102349-NPC RNA-seq cohort **(H)**, TCGA-CESC cohort **(I)**, TCGA-COAD cohort **(J)**, TCGA-READ cohort **(K)**, TCGA-BRCA cohort **(L)**, TCGA-SKCM cohort **(M)**, and TCGA-KIRC cohort **(N)**. **(O-U)** Scatter plots showing Spearman’s correlation between the apCAFs gene signature scores and CD4+ effector T cells gene signature scores in GSE102349-NPC RNA-seq cohort **(O)**, TCGA-CESC cohort **(P)**, TCGA-COAD cohort **(Q)**, TCGA-READ cohort **(R)**, TCGA-BRCA cohort **(S)**, TCGA-SKCM cohort **(T)**, and TCGA-KIRC cohort **(U)**. P-values were calculated by the log-rank test **(A-G)**. NPC, Nasopharyngeal Carcinoma; apCAFs, antigen-presenting CAFs.

Supplementary Figure 8 Spearman’s correlation between the apCAFs signature scores and CD4+ effector T cells signature scores in scRNA-seq datasets, as well as the expression level of immune checkpoint receptors in fibroblast subpopulations. **(A-G)** Scatter plots showing Spearman’s correlation between the apCAFs signature scores and CD4+ effector T cells signature scores in NPC **(A)**, BRCA **(B)**, AM (**C**), CM (**D**), RCC (**E**), CC (**F**), and CRC (**G**). **(H-P)** Dot plots showing the expression levels of CTLA4, LAG3, and TIGIT in distinct fibroblasts subpopulations of HNSCC **(H)**, OV **(I)**, NPC **(J)**, CC **(K)**, CRC **(L)**, BRCA **(M)**, AM **(N)**, CM **(O)**, and RCC **(P)**. Dot size indicates fraction of expressing cells, colored based on normalized expression levels **(H-P)**. HNSCC, Head and Neck Squamous Cell Carcinoma; OV, Ovarian Cancer; NPC, Nasopharyngeal Carcinoma; CC, Cervical Cancer; CRC, Colorectal Cancer; BRCA, Breast Cancer; AM, Acral Melanoma; CM, Cutaneous Melanoma; RCC, Renal Cell Carcinoma; apCAFs, antigen-presenting CAFs; myCAFs, myofibroblastic CAFs; eCAFs, extracellular matrix CAFs; iCAFs, inflammatory CAFs; NFs, normal fibroblasts.

Supplementary Figure 9 Immune characteristics of apCAFs. **(A-E)** Dot plots showing the expression levels of C1Q molecules in distinct fibroblasts subpopulations of NPC **(A)**, CC **(B)**, CRC **(C)**, BRCA **(D)**, and AM **(E)**. **(F-G)** Bar plots showing the selected signaling pathways with significant enrichment of GO: BP and HALLMARK terms for apCAFs compared to NFs in NPC **(F)** and RCC **(G)**. Differences in pathway activities scored per cell by GSVA between apCAFs and NFs. t values from a linear model, corrected for sample of origin. **(H-N)** Dot plots showing the expression profiles of molecule machinery involved in antigen processing and presentation in distinct fibroblasts subpopulations of NPC **(H)**, OV **(I)**, CRC **(J)**, BRCA **(K)**, AM **(L)**, CM **(M)** and RCC **(N)**. Dot size indicates fraction of expressing cells, colored based on normalized expression levels **(A-E, H-N)**. OV, Ovarian Cancer; NPC, Nasopharyngeal Carcinoma; CC, Cervical Cancer; CRC, Colorectal Cancer; BRCA, Breast Cancer; AM, Acral Melanoma; CM, Cutaneous Melanoma; RCC, Renal Cell Carcinoma; apCAFs, antigen-presenting CAFs; myCAFs, myofibroblastic CAFs; eCAFs, extracellular matrix CAFs; iCAFs, inflammatory CAFs; NFs, normal fibroblasts.

Supplementary Figure 10 Illustration of the spatial transcriptomic spots of HNSCC with apCAFs, tumor cells and CD4+ effector T cells signatures enrichment. **(A, F)** Left: Spatial transcriptomic spots with tumor cells signature enrichment in HNSCC201125T10 slice **(A)** and P210325T3 slice **(F)** of HNSCC; Right: Spatial transcriptomic spots with apCAFs signature enrichment in HNSCC201125T10 slice **(A)** and P210325T3 slice **(F)** of HNSCC. **(B, G)** Spatial transcriptomic spots with apCAFs and tumor cells signatures enrichment in one single plot in HNSCC201125T10 slice **(B)** and P210325T3 slice **(G)** of HNSCC. **(D, I)** Left: Spatial transcriptomic spots with CD4+ effector T cells gene signature enrichment in HNSCC201125T10 slice **(D)** and P210325T3 slice **(I)** of HNSCC; Right: Spatial transcriptomic spots with apCAFs gene signature enrichment in HNSCC201125T10 slice **(D)** and P210325T3 slice **(I)** of HNSCC. **(C, E, H, J)** Scatter plots showing Spearman’s correlation between apCAFs signature scores and both tumor cell signature scores and CD4+ effector T cell signature scores in the spatial transcriptomic spots in HNSCC201125T10 slice **(C, E)** and P210325T3 slice **(H, J)** of HNSCC. HNSCC, Head and Neck Squamous Cell Carcinoma; apCAFs, antigen-presenting CAFs.

Supplementary Figure 11 Illustration of the spatial transcriptomic spots of OV with apCAFs, tumor cells and CD4+ effector T cells signatures enrichment. **(A, F)** Left: Spatial transcriptomic spots with tumor cells signature enrichment in GSM6592135 slice **(A)** and GSM6592137 slice **(F)** of OV; Right: Spatial transcriptomic spots with apCAFs signature enrichment in GSM6592135 slice **(A)** and GSM6592137 slice **(F)** of OV. (**B**, **G)** Spatial transcriptomic spots with apCAFs and tumor cells signatures enrichment in one single plot in GSM6592135 slice **(B)** and GSM6592137 slice **(G)** of OV. **(D, I)** Left: Spatial transcriptomic spots with CD4+ effector T cells gene signature enrichment in GSM6592135 slice **(D)** and GSM6592137 slice **(I)** of OV; Right: Spatial transcriptomic spots with apCAFs gene signature enrichment in GSM6592135 slice **(D)** and GSM6592137 slice **(I)** of OV. **(C, E, H, J)** Scatter plots showing Spearman’s correlation between apCAFs signature scores and both tumor cell signature scores and CD4+ effector T cell signature scores in the spatial transcriptomic spots in GSM6592135 slice **(C, E)** and GSM6592137 slice **(H, J)** of OV. OV, Ovarian Cancer; apCAFs, antigen-presenting CAFs.

Supplementary Figure 12 Illustration of the spatial transcriptomic spots of BRCA with apCAFs, tumor cells and CD4+ effector T cells signatures enrichment. **(A, F, K)** Left: Spatial transcriptomic spots with tumor cells signature enrichment in CID4465 slice **(A)**, CID4535 slice **(F)** and CID44971 slice **(K)** of BRCA; Right: Spatial transcriptomic spots with apCAFs signature enrichment in CID4465 slice **(A)**, CID4535 slice **(F)** and CID44971 slice **(K)** of BRCA. **(B, G, L)** Spatial transcriptomic spots with apCAFs and tumor cells signatures enrichment in one single plot in CID4465 slice **(B)**, CID4535 slice **(G)** and CID44971 slice **(L)** of BRCA. **(D, I, N)** Left: Spatial transcriptomic spots with CD4+ effector T cells gene signature enrichment in CID4465 slice **(D)**, CID4535 slice **(I)** and CID44971 slice **(N)** of BRCA; Right: Spatial transcriptomic spots with apCAFs gene signature enrichment in CID4465 slice **(D)**, CID4535 slice **(I)** and CID44971 slice **(N)** of BRCA. **(C, E, H, J, M, O)** Scatter plots showing Spearman’s correlation between apCAFs signature scores and both tumor cell signature scores and CD4+ effector T cell signature scores in the spatial transcriptomic spots in CID4465 slice **(C, E)**, CID4535 slice **(H, J)** and CID44971 slice **(M, O)** of BRCA. BRCA, Breast Cancer; apCAFs, antigen-presenting CAFs.

Supplementary Figure 13 Illustration of the spatial transcriptomic spots of CRC with apCAFs, tumor cells and CD4+ effector T cells signatures enrichment. **(A, F)** Left: Spatial transcriptomic spots with tumor cells signature enrichment in GSM7089855 slice **(A)** and GSM7089857 slice **(F)** of CRC; Right: Spatial transcriptomic spots with apCAFs signature enrichment in GSM7089855 slice **(A)** and GSM7089857 slice **(F)** of CRC. **(B, G)** Spatial transcriptomic spots with apCAFs and tumor cells signatures enrichment in one single plot in GSM7089855 slice **(B)** and GSM7089857 slice **(G)** of CRC. **(D, I)** Left: Spatial transcriptomic spots with CD4+ effector T cells gene signature enrichment in GSM7089855 slice **(D)** and GSM7089857 slice **(I)** of CRC; Right: Spatial transcriptomic spots with apCAFs gene signature enrichment in GSM7089855 slice **(D)** and GSM7089857 slice **(I)** of CRC. **(C, E, H, J)** Scatter plots showing Spearman’s correlation between apCAFs signature scores and both tumor cell signature scores and CD4+ effector T cell signature scores in the spatial transcriptomic spots in GSM7089855 slice **(C, E)** and GSM7089857 slice **(H, J)** of CRC. CRC, Colorectal Cancer; apCAFs, antigen-presenting CAFs.

Supplementary Figure 14 Characterization of apCAFs origin. **(A, E)** Transcriptomic similarity analyses among apCAFs, NFs, endothelial cells, various macrophages and various dendritic cells in NPC **(A)** and RCC **(E)**. The darker the blue, the stronger the positive correlation, and the darker the red, the stronger the negative correlation. The numbers in the circle represent the correlation coefficient R. **(B, F)** Left: The cell trajectory along the NFs-apCAFs path in NPC **(B)** and RCC **(F)**; Right: The pseudotime trajectory along the NFs-apCAFs path in NPC **(B)** and RCC **(F)**. **(C, G)** Density distribution of apCAFs and NFs along the pseudotime trajectory in NPC **(C)** and RCC **(G)**. **(D, H)** Dynamic variation in CD74 during pseudotime trajectory in NPC **(D)** and RCC **(H)**. NPC, Nasopharyngeal Carcinoma; RCC, Renal Cell Carcinoma; apCAFs, antigen-presenting CAFs; NFs, normal fibroblasts; cDC1, conventional type 1 dendritic cells; cDC2, conventional type 2 dendritic cells; LAMP3+ DCs, LAMP3+ dendritic cells.

Supplementary Table 1 The clinical and sample information of selected studies containing scRNA-seq data of HNSCC, OV, NPC, CC, CRC, BRCA, AM, CM and RCC, and spatial transcriptome data of HNSCC, OV, BRCA and CRC. HNSCC, Head and Neck Squamous Cell Carcinoma; OV, Ovarian Cancer; NPC, Nasopharyngeal Carcinoma; CC, Cervical Cancer; CRC, Colorectal Cancer; BRCA, Breast Cancer; AM, Acral Melanoma; CM, Cutaneous Melanoma; RCC, Renal Cell Carcinoma.

Supplementary Table 2 Differentially expressed genes from major cell types derived from various solid tumor types including HNSCC, OV, NPC, CC, CRC, BRCA, AM, CM, and RCC. A two-sided Wilcoxon signed-rank test with Bonferroni correction was used to assess statistical significance. HNSCC, Head and Neck Squamous Cell Carcinoma; OV, Ovarian Cancer; NPC, Nasopharyngeal Carcinoma; CC, Cervical Cancer; CRC, Colorectal Cancer; BRCA, Breast Cancer; AM, Acral Melanoma; CM, Cutaneous Melanoma; RCC, Renal Cell Carcinoma.

Supplementary Table 3 Differentially expressed genes in the sub-clustering analysis of CAFs populations originating from various solid tumor types, such as HNSCC, OV, NPC, CC, CRC, BRCA, AM, CM, and RCC. A two-sided Wilcoxon signed-rank test with Bonferroni correction was used to assess statistical significance. HNSCC, Head and Neck Squamous Cell Carcinoma; OV, Ovarian Cancer; NPC, Nasopharyngeal Carcinoma; CC, Cervical Cancer; CRC, Colorectal Cancer; BRCA, Breast Cancer; AM, Acral Melanoma; CM, Cutaneous Melanoma; RCC, Renal Cell Carcinoma.

Supplementary Table 4 The number of cells identified for each CAFs subpopulation in each solid tumor type.

Supplementary Table 5 Signature Matrix derived from scRNA-seq data of various solid tumor types including HNSCC, OV, NPC, CC, CRC, BRCA, CM, and RCC for CIBERSORTx mediated digital cytometry analysis. HNSCC, Head and Neck Squamous Cell Carcinoma; OV, Ovarian Cancer; NPC, Nasopharyngeal Carcinoma; CC, Cervical Cancer; CRC, Colorectal Cancer; BRCA, Breast Cancer; CM, Cutaneous Melanoma; RCC, Renal Cell Carcinoma.

Supplementary Table 6 CIBERSORTx results and patient survival information of various bulk RNA-seq data including TCGA-HNSC, TCGA-OV, GSE102349-NPC, TCGA-CESC, TCGA-COAD, TCGA-READ, TCGA-BRCA, TCGA-SKCM, and TCGA-KIRC.

Supplementary Table 7 Gene lists of CD4+ effector T cells gene signature and apCAFs gene signatures derived from scRNA-seq data of various solid tumor types including HNSCC, OV, NPC, CC, CRC, BRCA, AM, CM, and RCC. HNSCC, Head and Neck Squamous Cell Carcinoma; OV, Ovarian Cancer; NPC, Nasopharyngeal Carcinoma; CC, Cervical Cancer; CRC, Colorectal Cancer; BRCA, Breast Cancer; AM, Acral Melanoma; CM, Cutaneous Melanoma; RCC, Renal Cell Carcinoma.

Supplementary Table 8 GSVA results for gene signatures representing apCAFs and CD4+ effector T cells were computed using various bulk RNA-seq data including TCGA-HNSC, TCGA-OV, GSE102349-NPC, TCGA-CESC, TCGA-COAD, TCGA-READ, TCGA-BRCA, TCGA-SKCM, and TCGA-KIRC.

Supplementary Table 9 The differential expression of the selected pathways of HALLMARK and GO: BP pathways between apCAFs and NFs were computed by the limma R package using scRNA-seq data from 4 solid tumor types including HNSCC, CC, NPC, and RCC. HNSCC, Head and Neck Squamous Cell Carcinoma; CC, Cervical Cancer; NPC, Nasopharyngeal Carcinoma; RCC, Renal Cell Carcinoma.

Supplementary Table 10 The semla R package was deployed for the purpose of inferring cell type proportions from spatial transcriptome data originating from 4 distinct solid tumor types, namely HNSCC, OV, BRCA, and CRC. HNSCC, Head and Neck Squamous Cell Carcinoma; OV, Ovarian Cancer; BRCA, Breast Cancer; CRC, Colorectal Cancer.

Supplementary Table 11 The AddModuleScore outcomes for gene signatures related to apCAFs and CD4+ effector T cells were obtained from various spatial transcriptome data, encompassing HNSCC, OV, BRCA, and CRC. HNSCC, Head and Neck Squamous Cell Carcinoma; OV, Ovarian Cancer; BRCA, Breast Cancer; CRC, Colorectal Cancer.

Supplementary Table 12 The top 5000 variably expressed genes in apCAFs, as well as their potential source cell types such as NFs, endothelial cells, different macrophages, and various dendritic cells, were identified using various scRNA-seq data encompassing HNSCC, CC, NPC, and RCC. HNSCC, Head and Neck Squamous Cell Carcinoma; CC, Cervical Cancer; NPC, Nasopharyngeal Carcinoma; RCC, Renal Cell Carcinoma.

Supplementary Table 13 AUCell AUC Z-score of regulons in fibroblasts subpopulations derived from scRNA-seq data of 6 solid tumor types including HNSCC, OV, NPC, BRCA, CM, and RCC. HNSCC, Head and Neck Squamous Cell Carcinoma; OV, Ovarian Cancer; NPC, Nasopharyngeal Carcinoma; BRCA, Breast Cancer; CM, Cutaneous Melanoma; RCC, Renal Cell Carcinoma.

Supplementary Table 14 GSVA results for HALLMARK_GLYCOLYSIS pathway in fibroblasts subpopulations derived from scRNA-seq data of 3 solid tumor types including HNSCC, NPC, and BRCA. HNSCC, Head and Neck Squamous Cell Carcinoma; NPC, Nasopharyngeal Carcinoma; BRCA, Breast Cancer.

Supplementary Table 15 The estimated metabolic flux from pyruvate to lactate in fibroblasts subpopulations derived from scRNA-seq data of 3 solid tumor types including HNSCC, NPC, and BRCA, using scFEA analysis in FLUXestimator website. HNSCC, Head and Neck Squamous Cell Carcinoma; NPC, Nasopharyngeal Carcinoma; BRCA, Breast Cancer.
