# Supplementary figures and images for: A pan-cancer single-cell transcriptional analysis of antigen-presenting cancer-associated fibroblasts in the tumor microenvironment
Source: Front Immunol. 2024 Jun 6;15:1372432. doi: 10.3389/fimmu.2024.1372432 (PMC11187094; doi:10.3389/fimmu.2024.1372432)

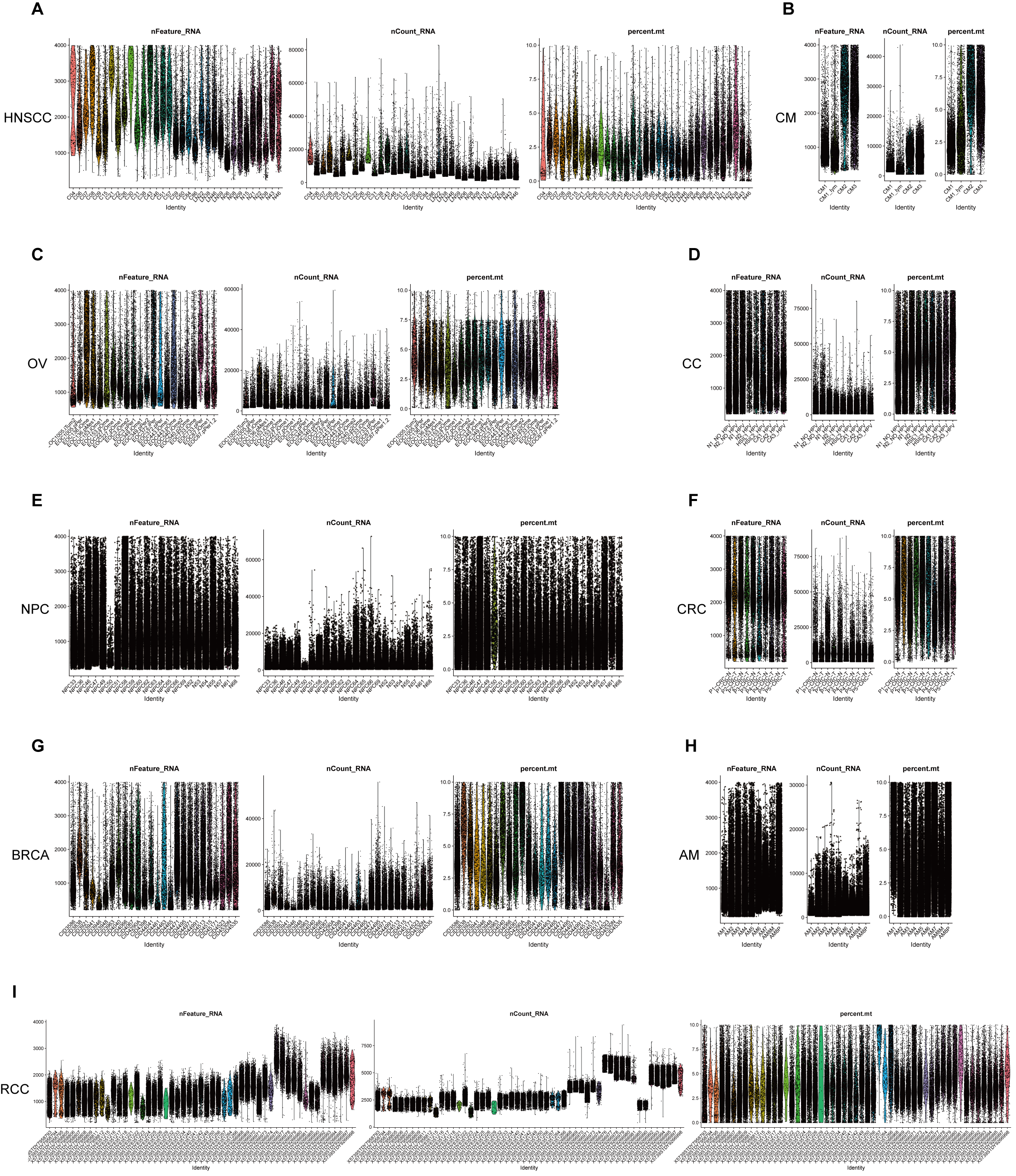

Supplement: Supplementary Figure 1 — Quality control of overall single-cell transcriptome profiles. (A-I) Violin plots of nFeature_RNA, nCount_RNA and percent.mt of all samples in HNSCC (A), CM (B), OV (C), CC (D), NPC (E), CRC (F), BRCA (G), AM (H) and RCC (I). The identities under the horizontal axis represent the source of the sample. HNSCC, Head and Neck Squamous Cell Carcinoma; CM, Cutaneous Melanoma; OV, Ovarian Cancer; CC, Cervical Cancer; NPC, Nasopharyngeal Carcinoma; CRC, Colorectal Cancer; BRCA, Breast Cancer; AM, Acral Melanoma; RCC, Renal Cell Carcinoma. [file Image_1.tif]

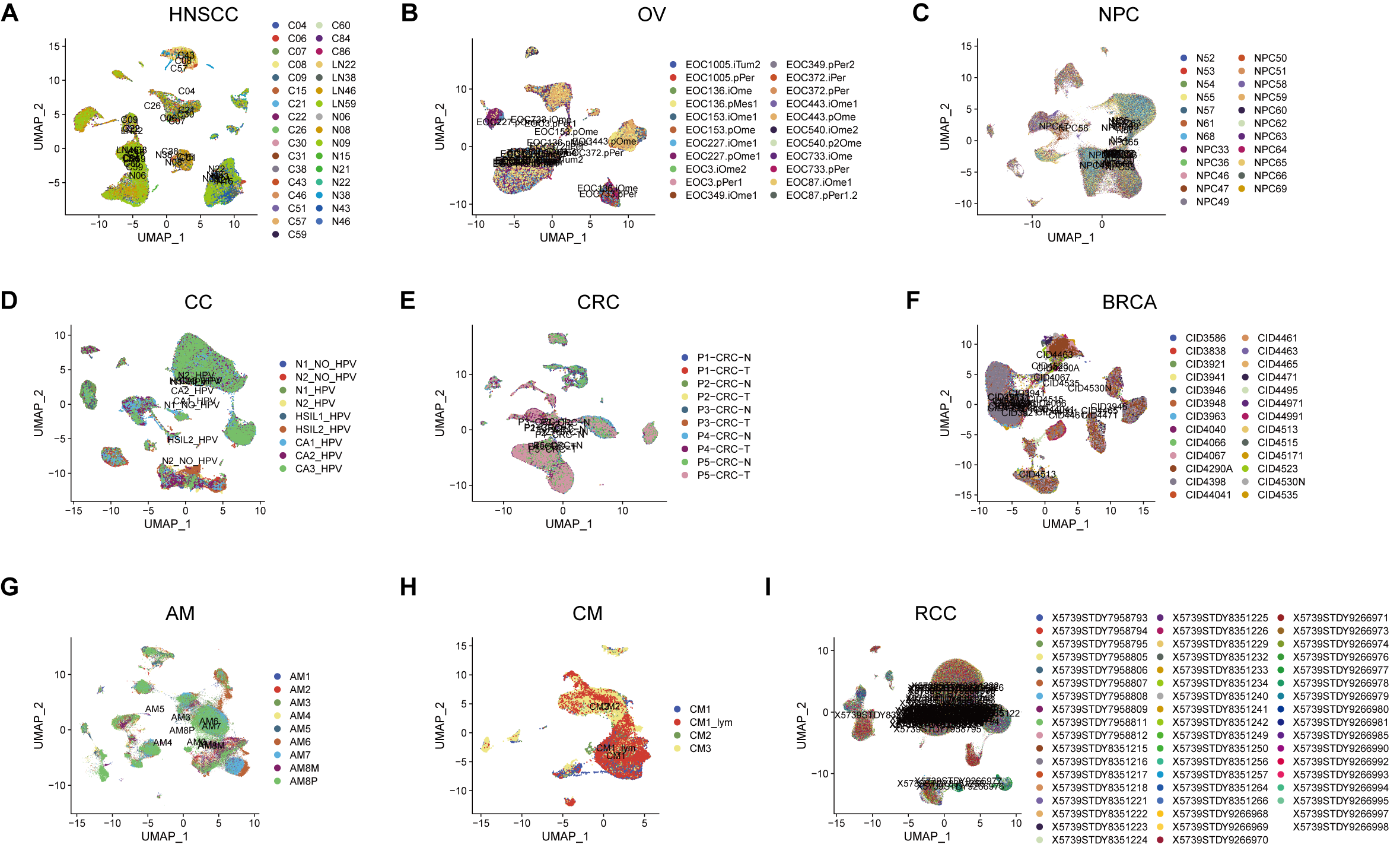

Supplement: Supplementary Figure 2 — Batch effects of overall single-cell transcriptome profiles. (A-I) UMAP plots of the overall 43507 cells in HNSCC (A), 46116 cells in OV (B), 236707 cells in NPC (C), 58964 cells in CC (D), 37345 cells in CRC (E), 77196 cells in BCRA (F), 181677 cells in AM (G), 23452 cells in CM (H) and 176664 cells in RCC (I), with each cell color coded for the sample of origin. HNSCC, Head and Neck Squamous Cell Carcinoma; OV, Ovarian Cancer; NPC, Nasopharyngeal Carcinoma; CC, Cervical Cancer; CRC, Colorectal Cancer; BRCA, Breast Cancer; AM, Acral Melanoma; CM, Cutaneous Melanoma; RCC, Renal Cell Carcinoma. [file Image_2.tif]

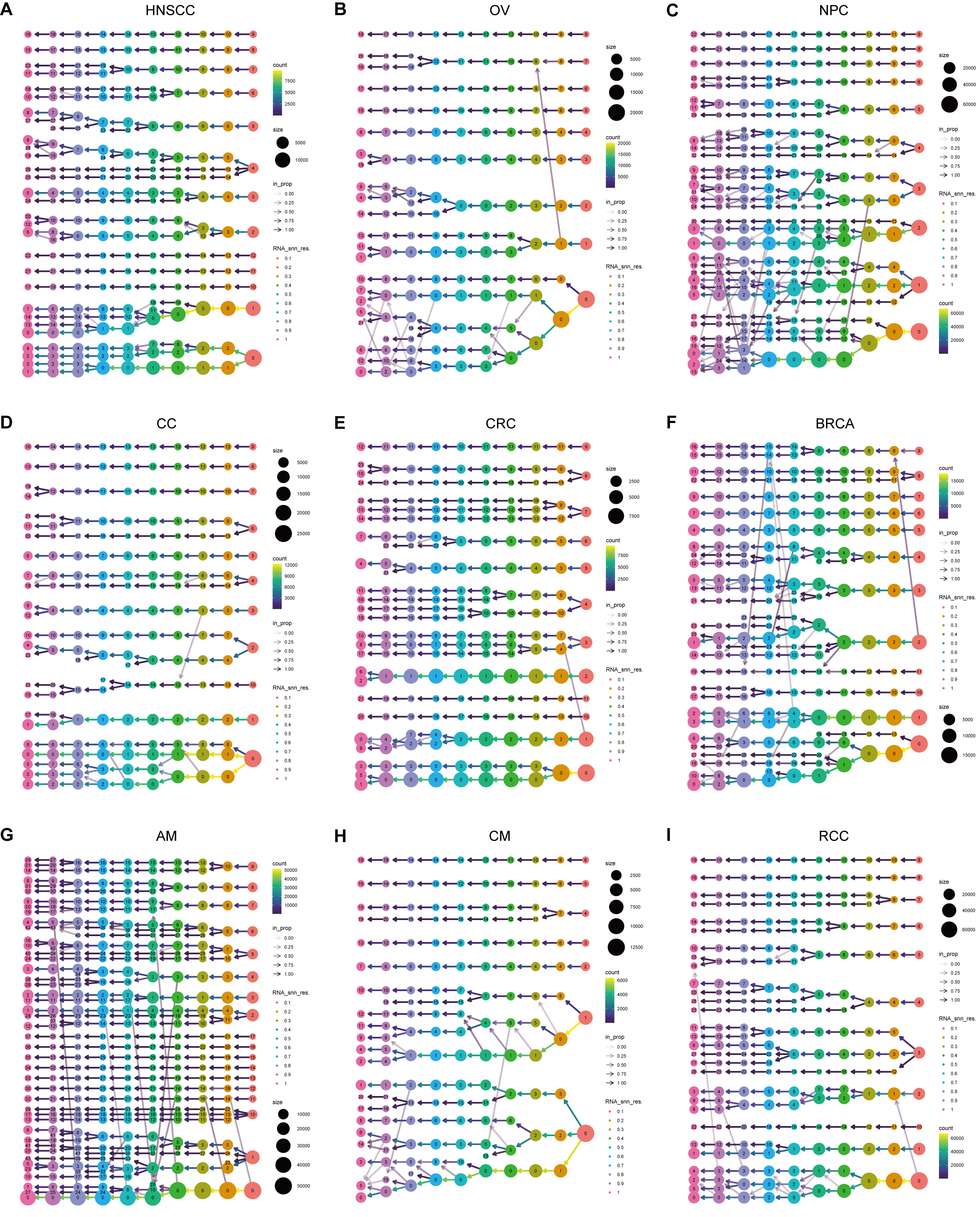

Supplement: Supplementary Figure 3 — Hierarchical clustering of overall single-cell transcriptome profiles. (A-I) Clustering trees showing hierarchical clustering for HNSCC (A), OV (B), NPC (C), CC (D), CRC (E), BRCA (F), AM (G), CM (H) and RCC (I). HNSCC, Head and Neck Squamous Cell Carcinoma; OV, Ovarian Cancer; NPC, Nasopharyngeal Carcinoma; CC, Cervical Cancer; CRC, Colorectal Cancer; BRCA, Breast Cancer; AM, Acral Melanoma; CM, Cutaneous Melanoma; RCC, Renal Cell Carcinoma. [file Image_3.tif]

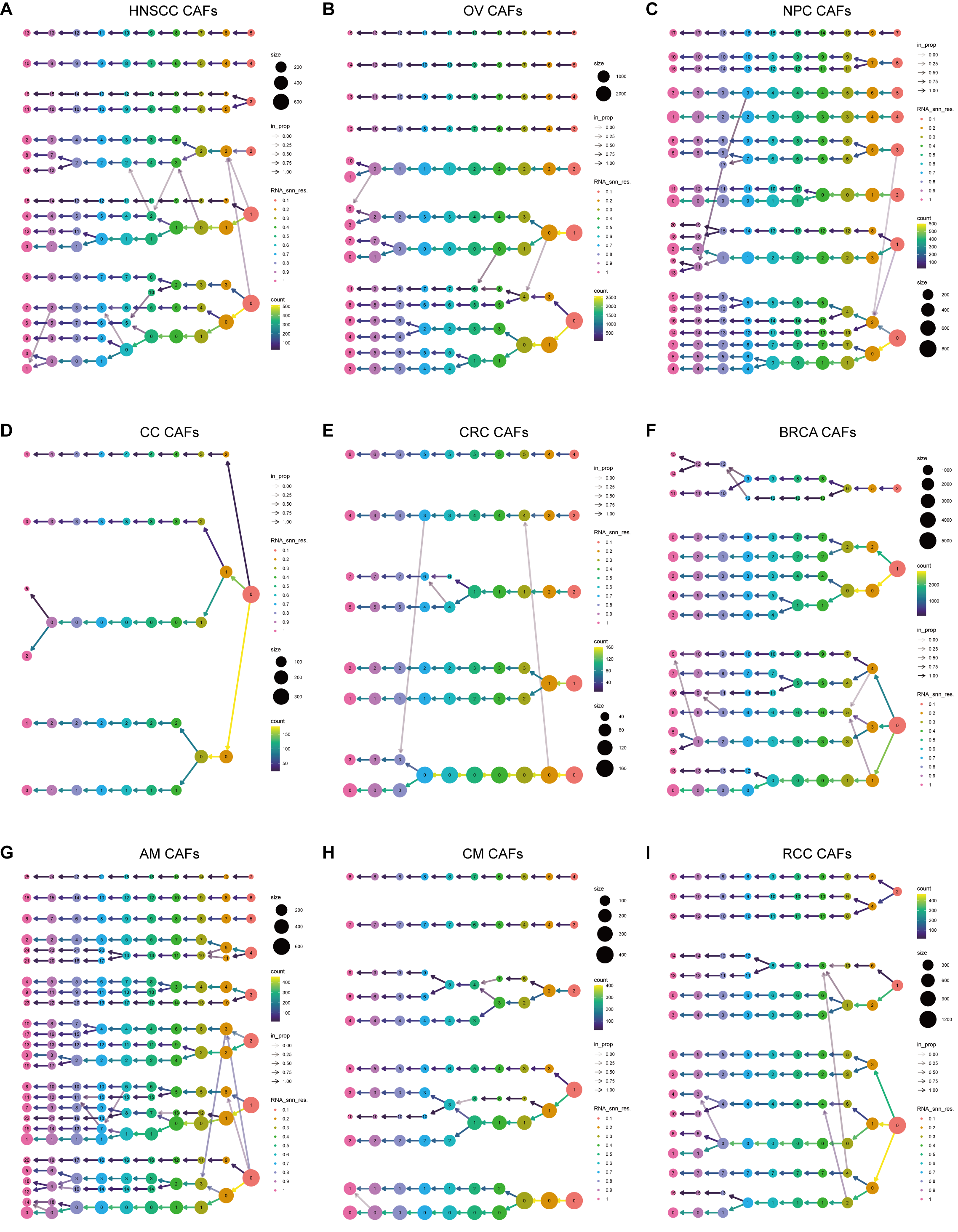

Supplement: Supplementary Figure 4 — Hierarchical clustering of overall CAFs single-cell transcriptome profiles. (A-I) Clustering trees showing hierarchical clustering for CAFs in HNSCC (A), OV (B), NPC (C), CC (D), CRC (E), BRCA (F), AM (G), CM (H) and RCC (I). HNSCC, Head and Neck Squamous Cell Carcinoma; OV, Ovarian Cancer; NPC, Nasopharyngeal Carcinoma; CC, Cervical Cancer; CRC, Colorectal Cancer; BRCA, Breast Cancer; AM, Acral Melanoma; CM, Cutaneous Melanoma; RCC, Renal Cell Carcinoma. [file Image_4.tif]

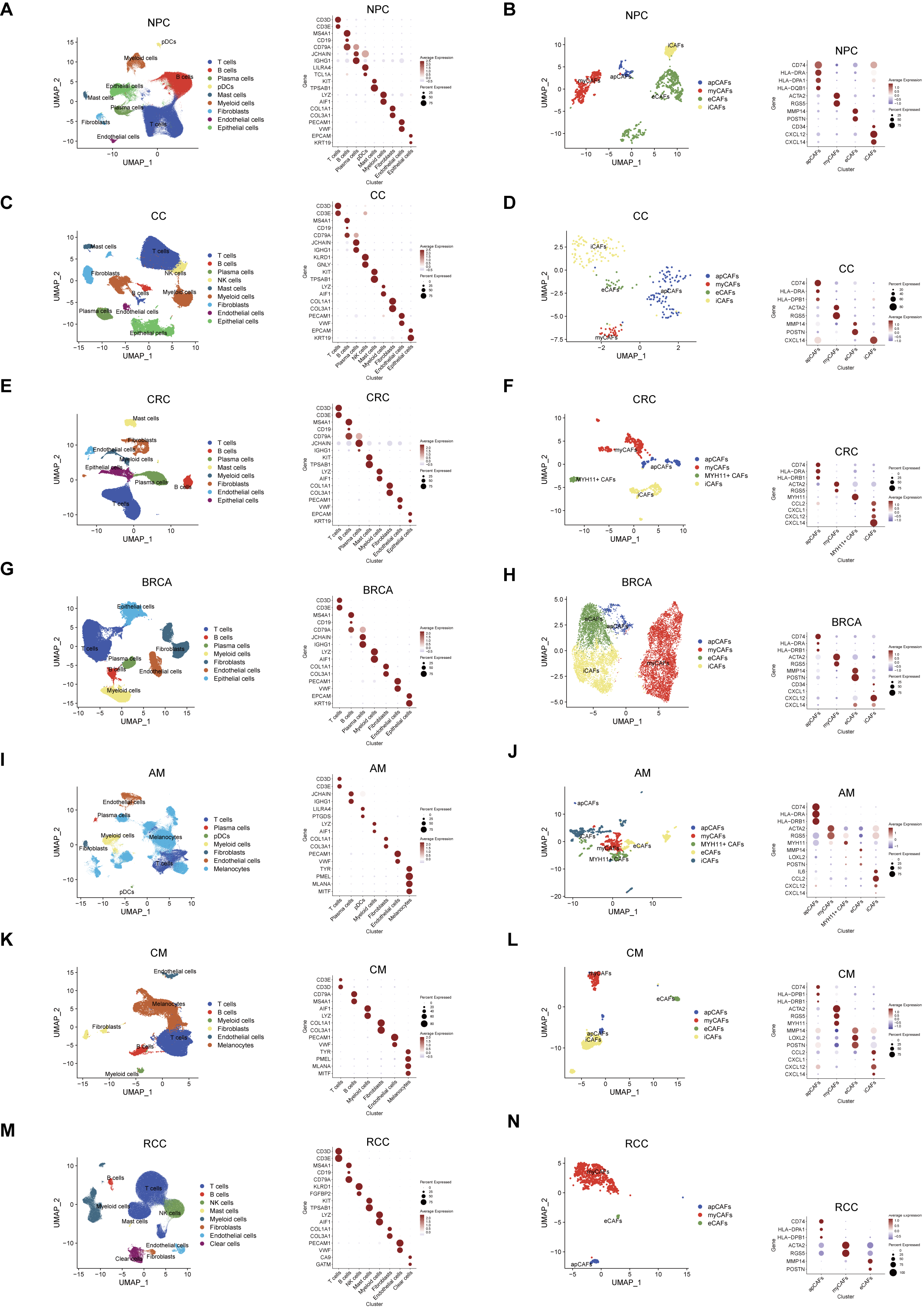

Supplement: Supplementary Figure 5 — Identification of apCAFs in various solid tumor types. (A, C, E, G, I, K, M) Left: UMAP plots showing the major cell types in NPC (A), CC (C), CRC (E), BRCA (G), AM (I), CM (K) and RCC (M); Right: Dot plots showing selected cell marker genes expression levels of the major cell types in NPC (A), CC (C), CRC (E), BRCA (G), AM (I), CM (K) and RCC (M). (B, D, F, H, J, L, N) Left: UMAP plots showing 4 major subpopulations of CAFs in NPC (B), CC (D), CRC (F), BRCA (H), 5 major subpopulations of CAFs in AM (J), 4 major subpopulations of CAFs in CM (L) and 3 major subpopulations of CAFs in RCC (N). Right: Dot plots showing selected cell marker genes expression levels for each subpopulation of CAFs in NPC (B), CC (D), CRC (F), BRCA (H), AM (J), CM (L) and RCC (N). Dot size indicates fraction of expressing cells, colored based on normalized expression levels (A-N: Right). NPC, Nasopharyngeal Carcinoma; CC, Cervical Cancer; CRC, Colorectal Cancer; BRCA, Breast Cancer; AM, Acral Melanoma; CM, Cutaneous Melanoma; RCC, Renal Cell Carcinoma; apCAFs, antigen-presenting CAFs; myCAFs, myofibroblastic CAFs; eCAFs, extracellular matrix CAFs; iCAFs, inflammatory CAFs, pDCs, plasmacytoid dendritic cells. [file Image_5.tif]

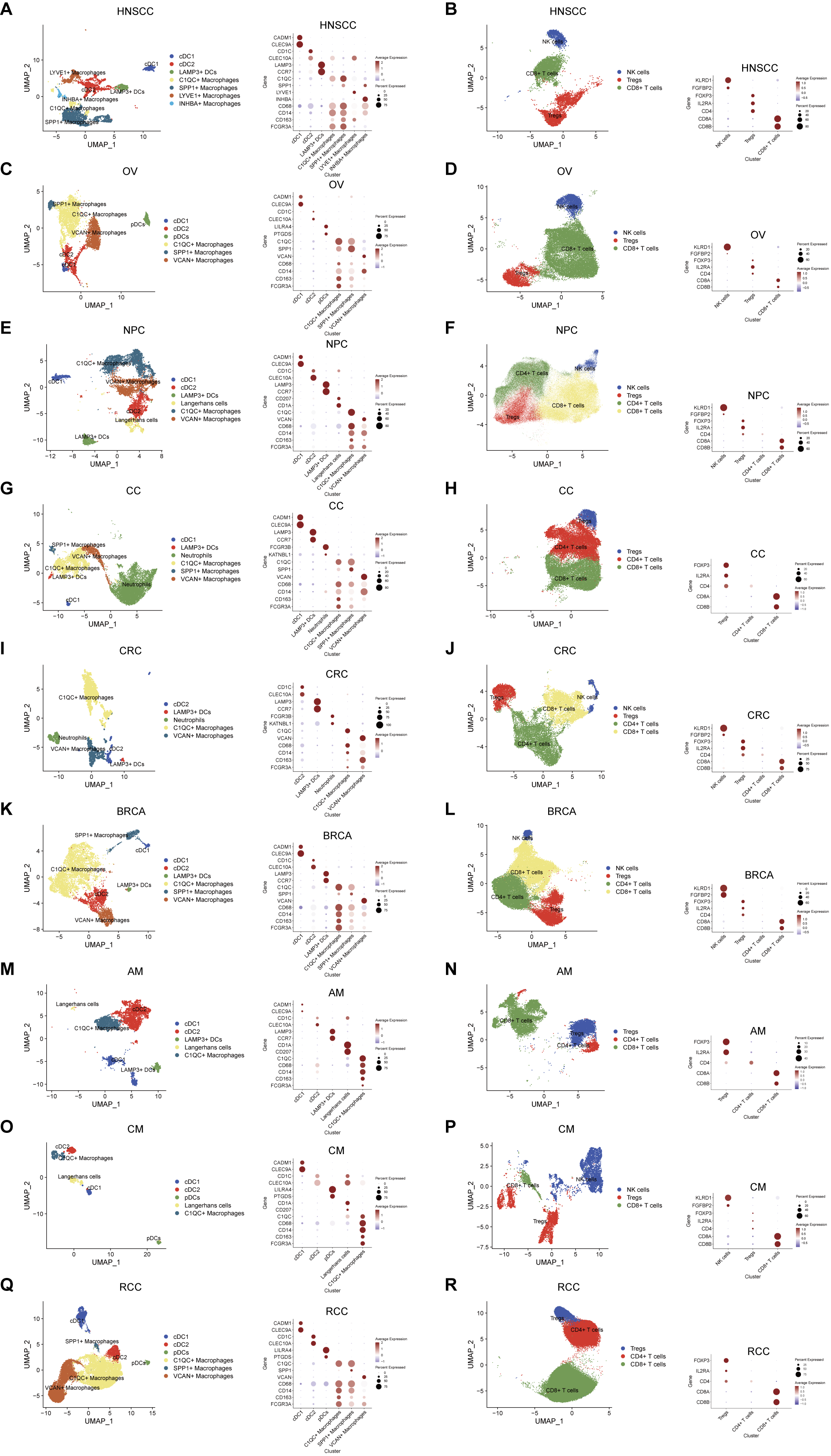

Supplement: Supplementary Figure 6 — Myeloid cells and T cells subpopulations in various solid tumor types. (A, C, E, G, I, K, M, O, Q) Left: UMAP plots showing 7 major subpopulations of myeloid cells in HNSCC (A), 6 major subpopulations of myeloid cells in OV (C), NPC (E), CC (G), 5 major subpopulations of myeloid cells in CRC (I), 6 major subpopulations of myeloid cells in BRCA (K), 5 major subpopulations of myeloid cells in AM (M), CM (O), and 6 major subpopulations of myeloid cells in RCC (Q). Right: Dot plots showing selected cell marker genes expression levels for the major subpopulations of myeloid cells in HNSCC (A), OV (C), NPC (E), CC (G), CRC (I), BRCA (K), AM (M), CM (O) and RCC (Q). (B, D, F, H, J, L, N, P, R) Left: UMAP plots showing 3 major subpopulations of T cells in HNSCC (B), OV (D), 4 major subpopulations of T cells in NPC (F), 3 major subpopulations of T cells in CC (H), 4 major subpopulations of T cells in CRC (J), BRCA (L), and 3 major subpopulations of T cells in AM (N), CM (P), RCC (R). Right: Dot plots showing selected cell marker genes expression levels for the major subpopulations of T cells in HNSCC (B), OV (D), NPC (F), CC (H), CRC (J), BRCA (L), AM (N), CM (P) and RCC (R). Dot size indicates fraction of expressing cells, colored based on normalized expression levels (A-R: Right). HNSCC, Head and Neck Squamous Cell Carcinoma; OV, Ovarian Cancer; NPC, Nasopharyngeal Carcinoma; CC, Cervical Cancer; CRC, Colorectal Cancer; BRCA, Breast Cancer; AM, Acral Melanoma; CM, Cutaneous Melanoma; RCC, Renal Cell Carcinoma; cDC1, conventional type 1 dendritic cells; cDC2, conventional type 2 dendritic cells; LAMP3+ DCs, LAMP3+ dendritic cells; pDCs, plasmacytoid dendritic cells; Tregs, regulatory T cells. [file Image_6.tif]

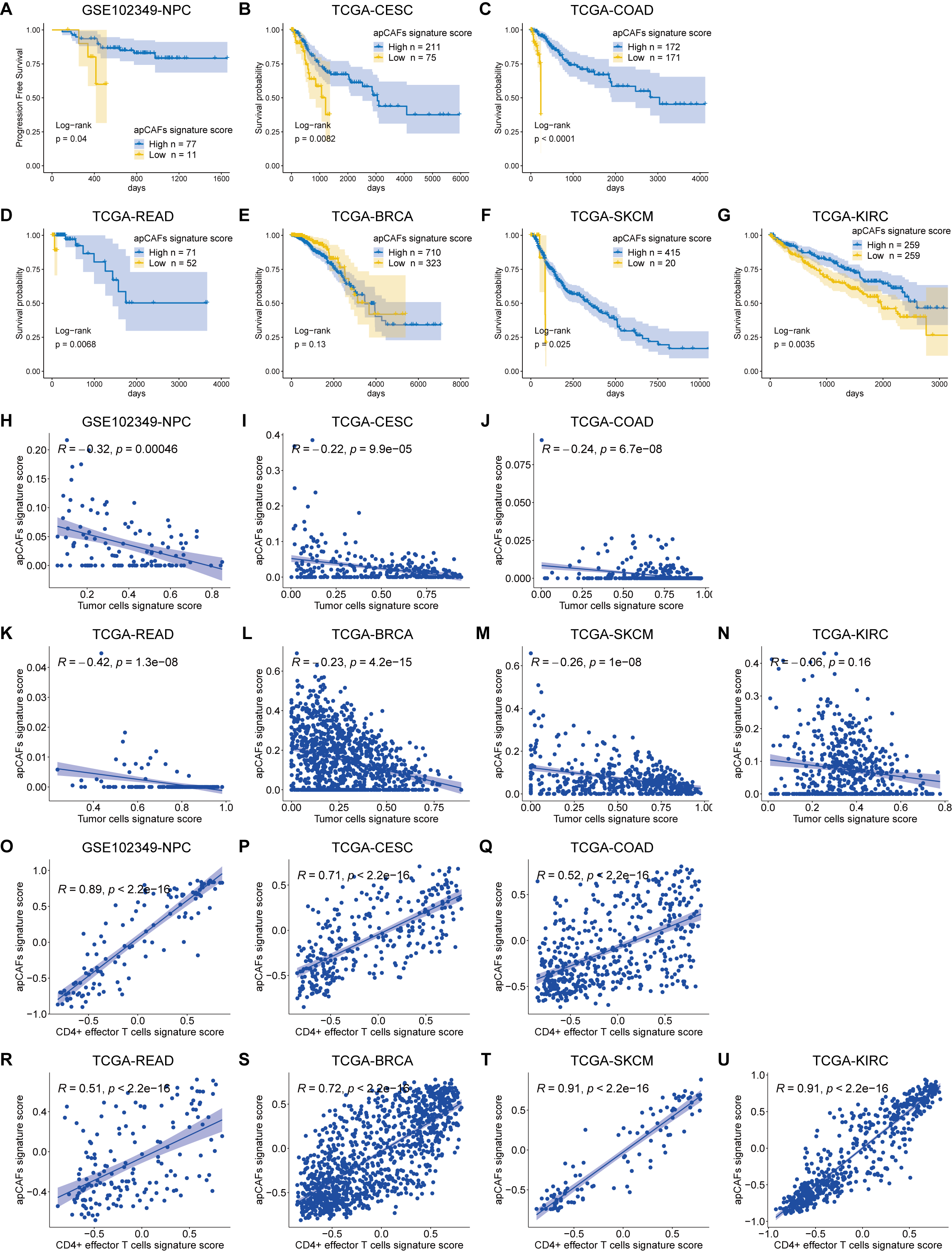

Supplement: Supplementary Figure 7 — apCAFs show the anti-tumor effects. (A) Kaplan-Meier plot showing progression free survival rate of patients by apCAFs signature scores in GSE102349-NPC RNA-seq cohort. (B-G) Kaplan-Meier plots showing overall survival probability of patients by apCAFs signature scores in TCGA-CESC cohort (B), TCGA-COAD cohort (C), TCGA-READ cohort (D), TCGA-BRCA cohort (E), TCGA-SKCM cohort (F) and TCGA-KIRC cohort (G). (H-N) Scatter plots showing Spearman’s correlation between the apCAFs signature scores and tumor cells signature scores in GSE102349-NPC RNA-seq cohort (H), TCGA-CESC cohort (I), TCGA-COAD cohort (J), TCGA-READ cohort (K), TCGA-BRCA cohort (L), TCGA-SKCM cohort (M), and TCGA-KIRC cohort (N). (O-U) Scatter plots showing Spearman’s correlation between the apCAFs gene signature scores and CD4+ effector T cells gene signature scores in GSE102349-NPC RNA-seq cohort (O), TCGA-CESC cohort (P), TCGA-COAD cohort (Q), TCGA-READ cohort (R), TCGA-BRCA cohort (S), TCGA-SKCM cohort (T), and TCGA-KIRC cohort (U). P-values were calculated by the log-rank test (A-G). NPC, Nasopharyngeal Carcinoma; apCAFs, antigen-presenting CAFs. [file Image_7.tif]

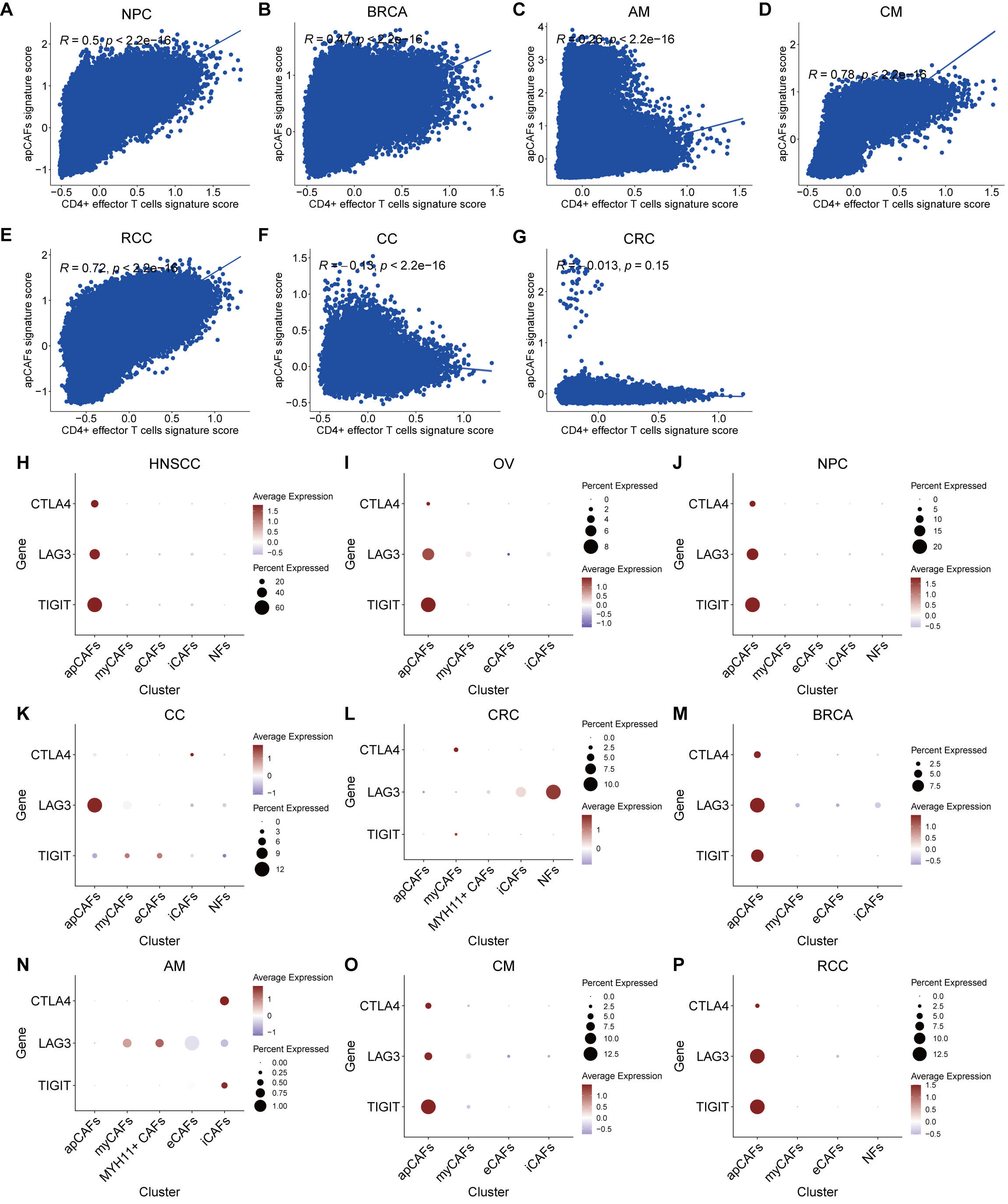

Supplement: Supplementary Figure 8 — Spearman’s correlation between the apCAFs signature scores and CD4+ effector T cells signature scores in scRNA-seq datasets, as well as the expression level of immune checkpoint receptors in fibroblast subpopulations. (A-G) Scatter plots showing Spearman’s correlation between the apCAFs signature scores and CD4+ effector T cells signature scores in NPC (A), BRCA (B), AM (C), CM (D), RCC (E), CC (F), and CRC (G). (H-P) Dot plots showing the expression levels of CTLA4, LAG3, and TIGIT in distinct fibroblasts subpopulations of HNSCC (H), OV (I), NPC (J), CC (K), CRC (L), BRCA (M), AM (N), CM (O), and RCC (P). Dot size indicates fraction of expressing cells, colored based on normalized expression levels (H-P). HNSCC, Head and Neck Squamous Cell Carcinoma; OV, Ovarian Cancer; NPC, Nasopharyngeal Carcinoma; CC, Cervical Cancer; CRC, Colorectal Cancer; BRCA, Breast Cancer; AM, Acral Melanoma; CM, Cutaneous Melanoma; RCC, Renal Cell Carcinoma; apCAFs, antigen-presenting CAFs; myCAFs, myofibroblastic CAFs; eCAFs, extracellular matrix CAFs; iCAFs, inflammatory CAFs; NFs, normal fibroblasts. [file Image_8.tif]

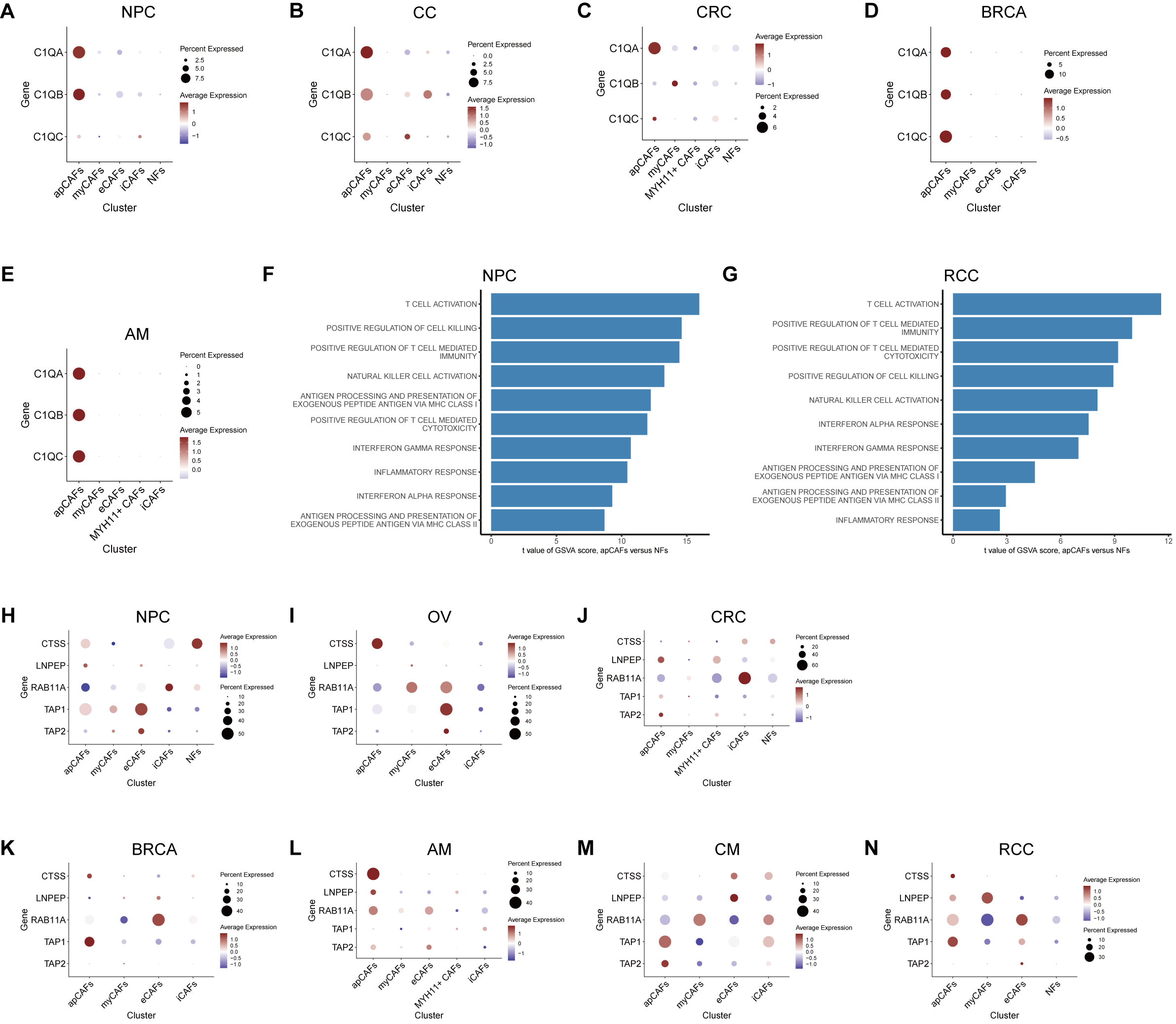

Supplement: Supplementary Figure 9 — Immune characteristics of apCAFs. (A-E) Dot plots showing the expression levels of C1Q molecules in distinct fibroblasts subpopulations of NPC (A), CC (B), CRC (C), BRCA (D), and AM (E). (F-G) Bar plots showing the selected signaling pathways with significant enrichment of GO: BP and HALLMARK terms for apCAFs compared to NFs in NPC (F) and RCC (G). Differences in pathway activities scored per cell by GSVA between apCAFs and NFs. t values from a linear model, corrected for sample of origin. (H-N) Dot plots showing the expression profiles of molecule machinery involved in antigen processing and presentation in distinct fibroblasts subpopulations of NPC (H), OV (I), CRC (J), BRCA (K), AM (L), CM (M) and RCC (N). Dot size indicates fraction of expressing cells, colored based on normalized expression levels (A-E, H-N). OV, Ovarian Cancer; NPC, Nasopharyngeal Carcinoma; CC, Cervical Cancer; CRC, Colorectal Cancer; BRCA, Breast Cancer; AM, Acral Melanoma; CM, Cutaneous Melanoma; RCC, Renal Cell Carcinoma; apCAFs, antigen-presenting CAFs; myCAFs, myofibroblastic CAFs; eCAFs, extracellular matrix CAFs; iCAFs, inflammatory CAFs; NFs, normal fibroblasts. [file Image_9.tif]

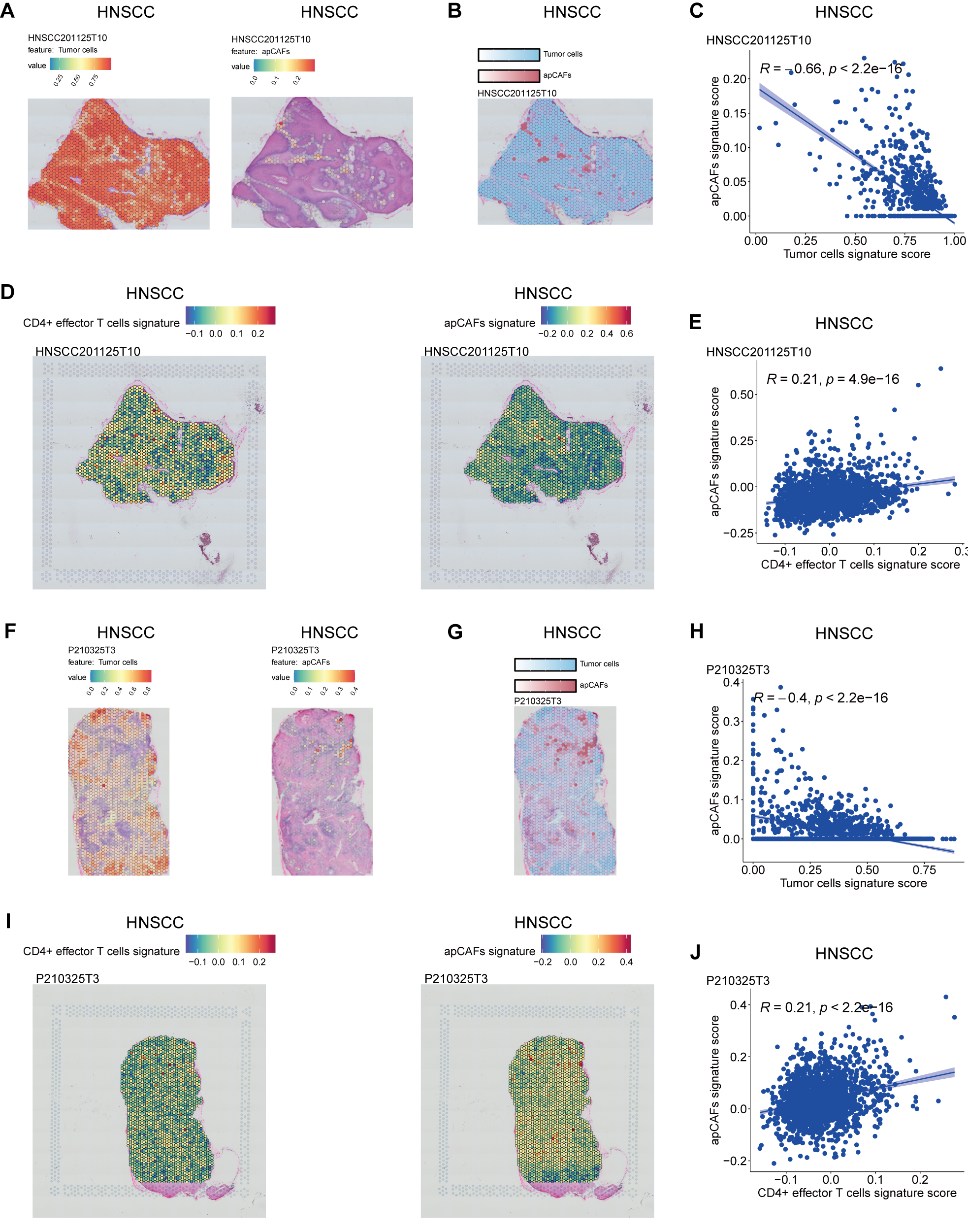

Supplement: Supplementary Figure 10 — Illustration of the spatial transcriptomic spots of HNSCC with apCAFs, tumor cells and CD4+ effector T cells signatures enrichment. (A, F) Left: Spatial transcriptomic spots with tumor cells signature enrichment in HNSCC201125T10 slice (A) and P210325T3 slice (F) of HNSCC; Right: Spatial transcriptomic spots with apCAFs signature enrichment in HNSCC201125T10 slice (A) and P210325T3 slice (F) of HNSCC. (B, G) Spatial transcriptomic spots with apCAFs and tumor cells signatures enrichment in one single plot in HNSCC201125T10 slice (B) and P210325T3 slice (G) of HNSCC. (D, I) Left: Spatial transcriptomic spots with CD4+ effector T cells gene signature enrichment in HNSCC201125T10 slice (D) and P210325T3 slice (I) of HNSCC; Right: Spatial transcriptomic spots with apCAFs gene signature enrichment in HNSCC201125T10 slice (D) and P210325T3 slice (I) of HNSCC. (C, E, H, J) Scatter plots showing Spearman’s correlation between apCAFs signature scores and both tumor cell signature scores and CD4+ effector T cell signature scores in the spatial transcriptomic spots in HNSCC201125T10 slice (C, E) and P210325T3 slice (H, J) of HNSCC. HNSCC, Head and Neck Squamous Cell Carcinoma; apCAFs, antigen-presenting CAFs. [file Image_10.tif]

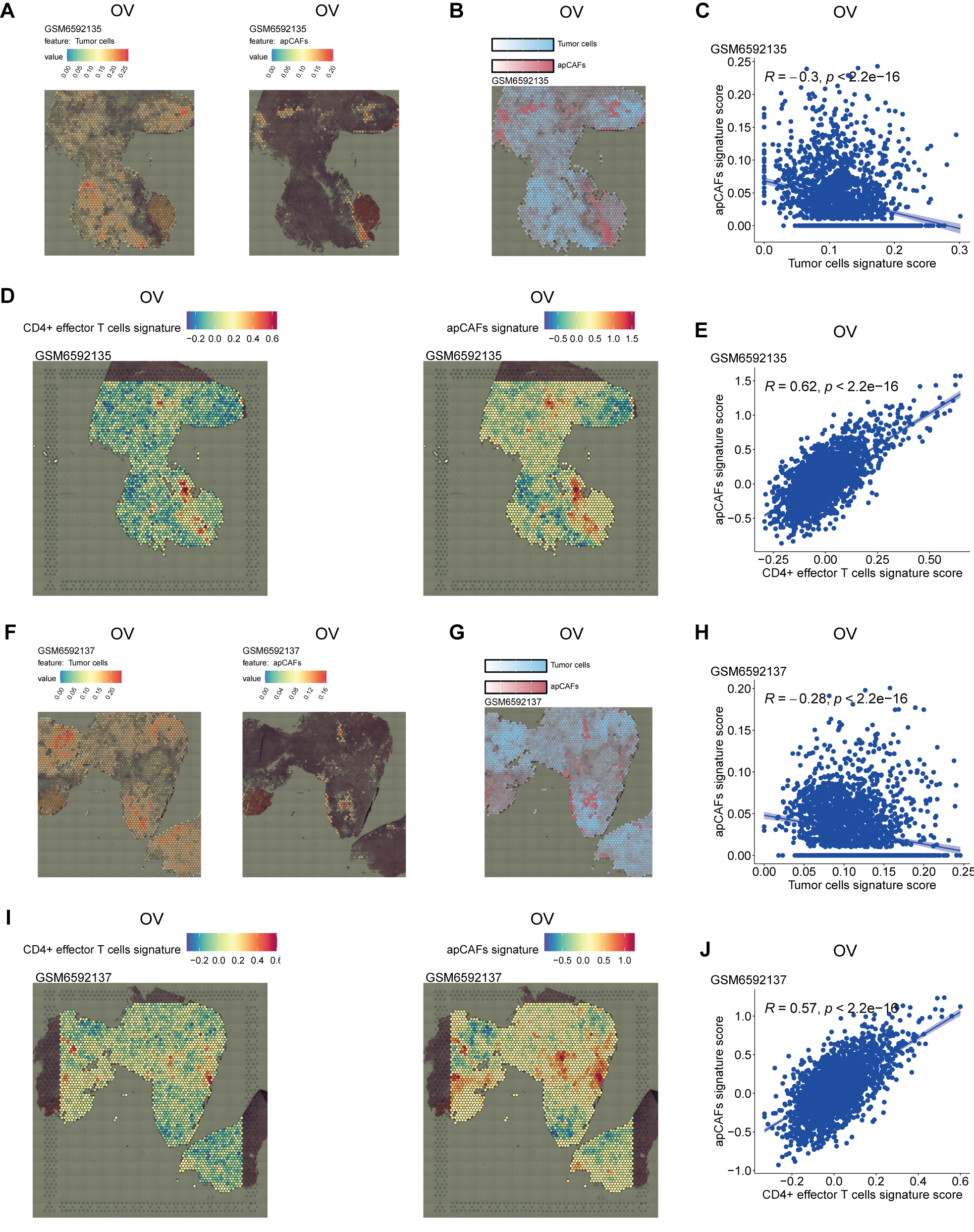

Supplement: Supplementary Figure 11 — Illustration of the spatial transcriptomic spots of OV with apCAFs, tumor cells and CD4+ effector T cells signatures enrichment. (A, F) Left: Spatial transcriptomic spots with tumor cells signature enrichment in GSM6592135 slice (A) and GSM6592137 slice (F) of OV; Right: Spatial transcriptomic spots with apCAFs signature enrichment in GSM6592135 slice (A) and GSM6592137 slice (F) of OV. (B, G) Spatial transcriptomic spots with apCAFs and tumor cells signatures enrichment in one single plot in GSM6592135 slice (B) and GSM6592137 slice (G) of OV. (D, I) Left: Spatial transcriptomic spots with CD4+ effector T cells gene signature enrichment in GSM6592135 slice (D) and GSM6592137 slice (I) of OV; Right: Spatial transcriptomic spots with apCAFs gene signature enrichment in GSM6592135 slice (D) and GSM6592137 slice (I) of OV. (C, E, H, J) Scatter plots showing Spearman’s correlation between apCAFs signature scores and both tumor cell signature scores and CD4+ effector T cell signature scores in the spatial transcriptomic spots in GSM6592135 slice (C, E) and GSM6592137 slice (H, J) of OV. OV, Ovarian Cancer; apCAFs, antigen-presenting CAFs. [file Image_11.tif]

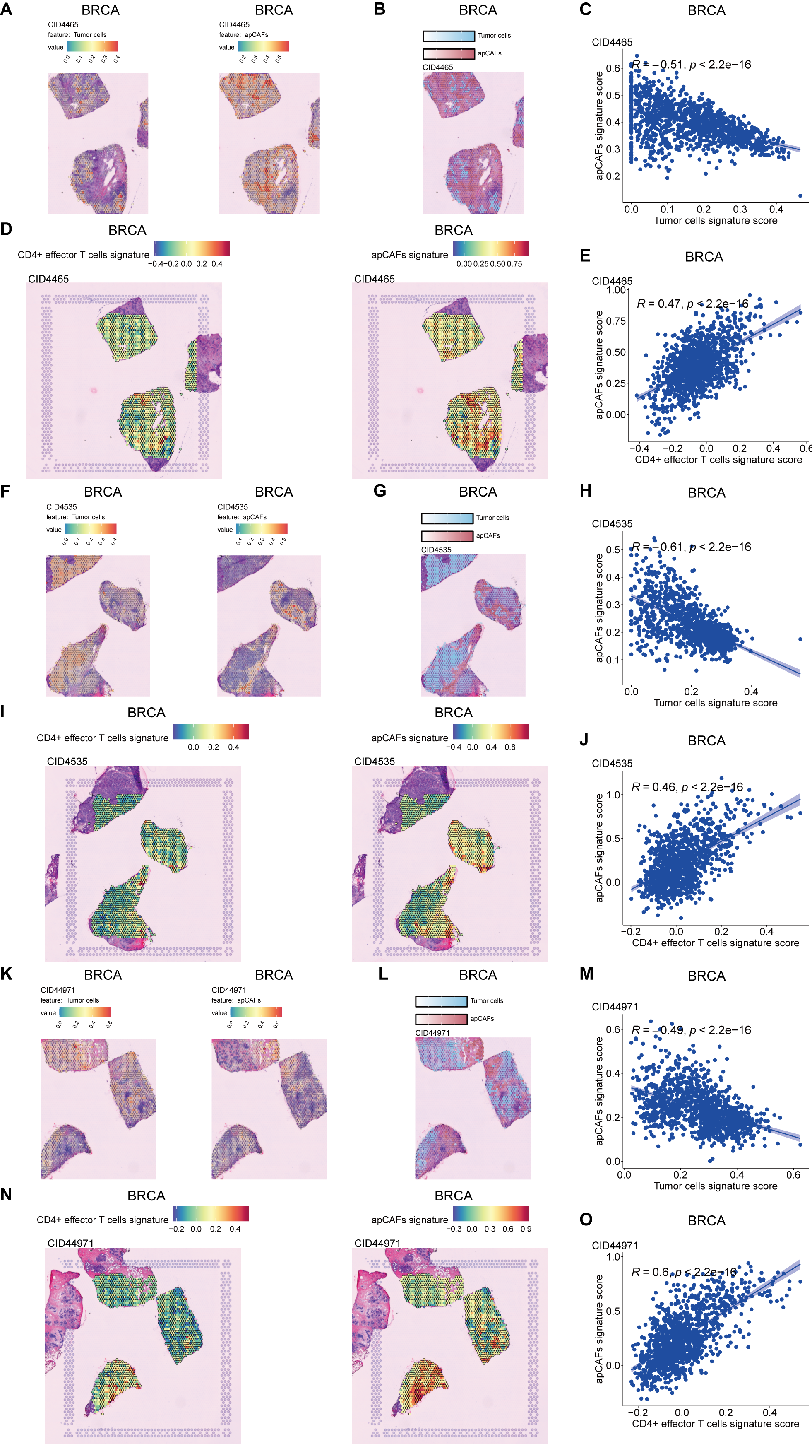

Supplement: Supplementary Figure 12 — Illustration of the spatial transcriptomic spots of BRCA with apCAFs, tumor cells and CD4+ effector T cells signatures enrichment. (A, F, K) Left: Spatial transcriptomic spots with tumor cells signature enrichment in CID4465 slice (A), CID4535 slice (F) and CID44971 slice (K) of BRCA; Right: Spatial transcriptomic spots with apCAFs signature enrichment in CID4465 slice (A), CID4535 slice (F) and CID44971 slice (K) of BRCA. (B, G, L) Spatial transcriptomic spots with apCAFs and tumor cells signatures enrichment in one single plot in CID4465 slice (B), CID4535 slice (G) and CID44971 slice (L) of BRCA. (D, I, N) Left: Spatial transcriptomic spots with CD4+ effector T cells gene signature enrichment in CID4465 slice (D), CID4535 slice (I) and CID44971 slice (N) of BRCA; Right: Spatial transcriptomic spots with apCAFs gene signature enrichment in CID4465 slice (D), CID4535 slice (I) and CID44971 slice (N) of BRCA. (C, E, H, J, M, O) Scatter plots showing Spearman’s correlation between apCAFs signature scores and both tumor cell signature scores and CD4+ effector T cell signature scores in the spatial transcriptomic spots in CID4465 slice (C, E), CID4535 slice (H, J) and CID44971 slice (M, O) of BRCA. BRCA, Breast Cancer; apCAFs, antigen-presenting CAFs. [file Image_12.tif]

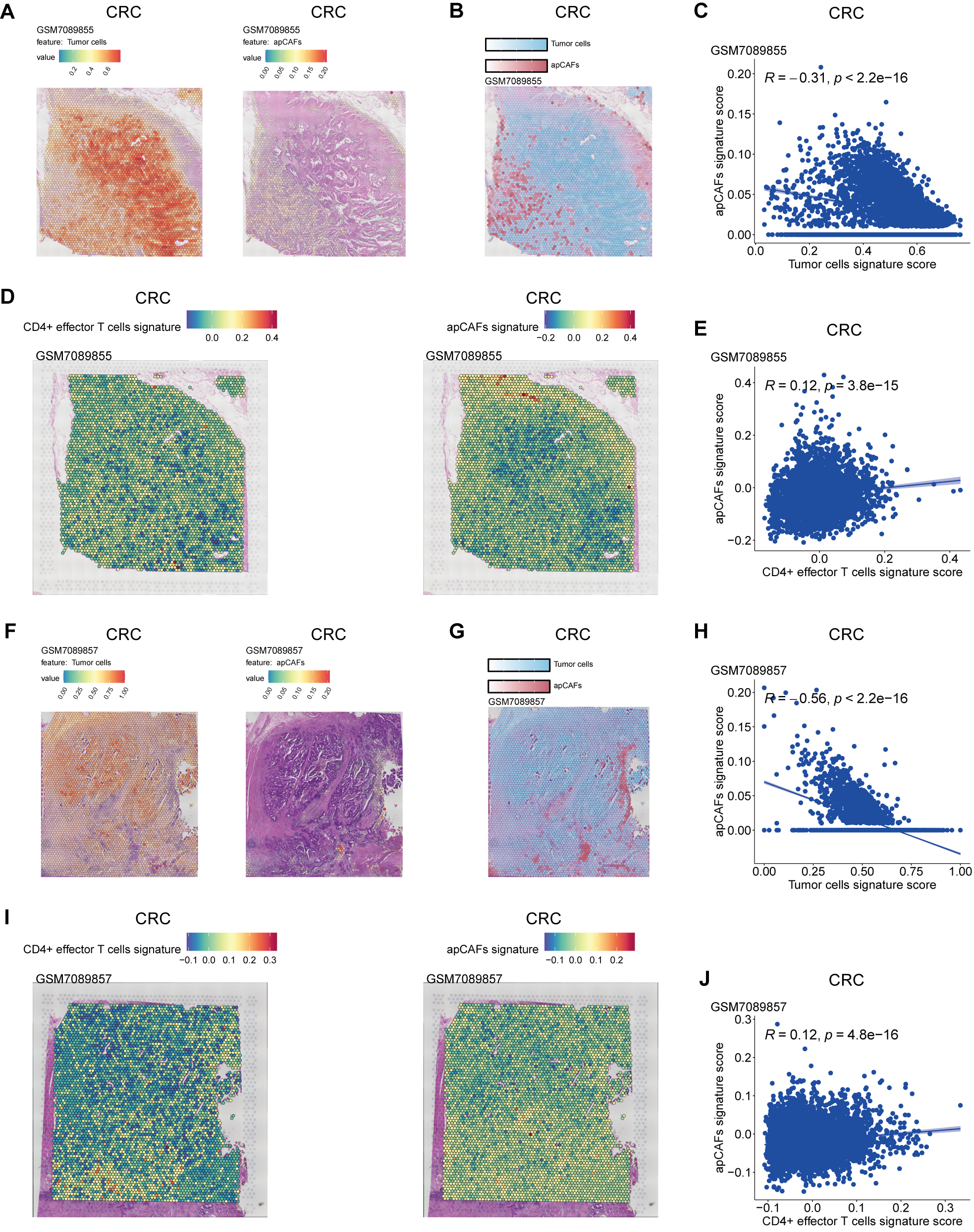

Supplement: Supplementary Figure 13 — Illustration of the spatial transcriptomic spots of CRC with apCAFs, tumor cells and CD4+ effector T cells signatures enrichment. (A, F) Left: Spatial transcriptomic spots with tumor cells signature enrichment in GSM7089855 slice (A) and GSM7089857 slice (F) of CRC; Right: Spatial transcriptomic spots with apCAFs signature enrichment in GSM7089855 slice (A) and GSM7089857 slice (F) of CRC. (B, G) Spatial transcriptomic spots with apCAFs and tumor cells signatures enrichment in one single plot in GSM7089855 slice (B) and GSM7089857 slice (G) of CRC. (D, I) Left: Spatial transcriptomic spots with CD4+ effector T cells gene signature enrichment in GSM7089855 slice (D) and GSM7089857 slice (I) of CRC; Right: Spatial transcriptomic spots with apCAFs gene signature enrichment in GSM7089855 slice (D) and GSM7089857 slice (I) of CRC. (C, E, H, J) Scatter plots showing Spearman’s correlation between apCAFs signature scores and both tumor cell signature scores and CD4+ effector T cell signature scores in the spatial transcriptomic spots in GSM7089855 slice (C, E) and GSM7089857 slice (H, J) of CRC. CRC, Colorectal Cancer; apCAFs, antigen-presenting CAFs. [file Image_13.tif]

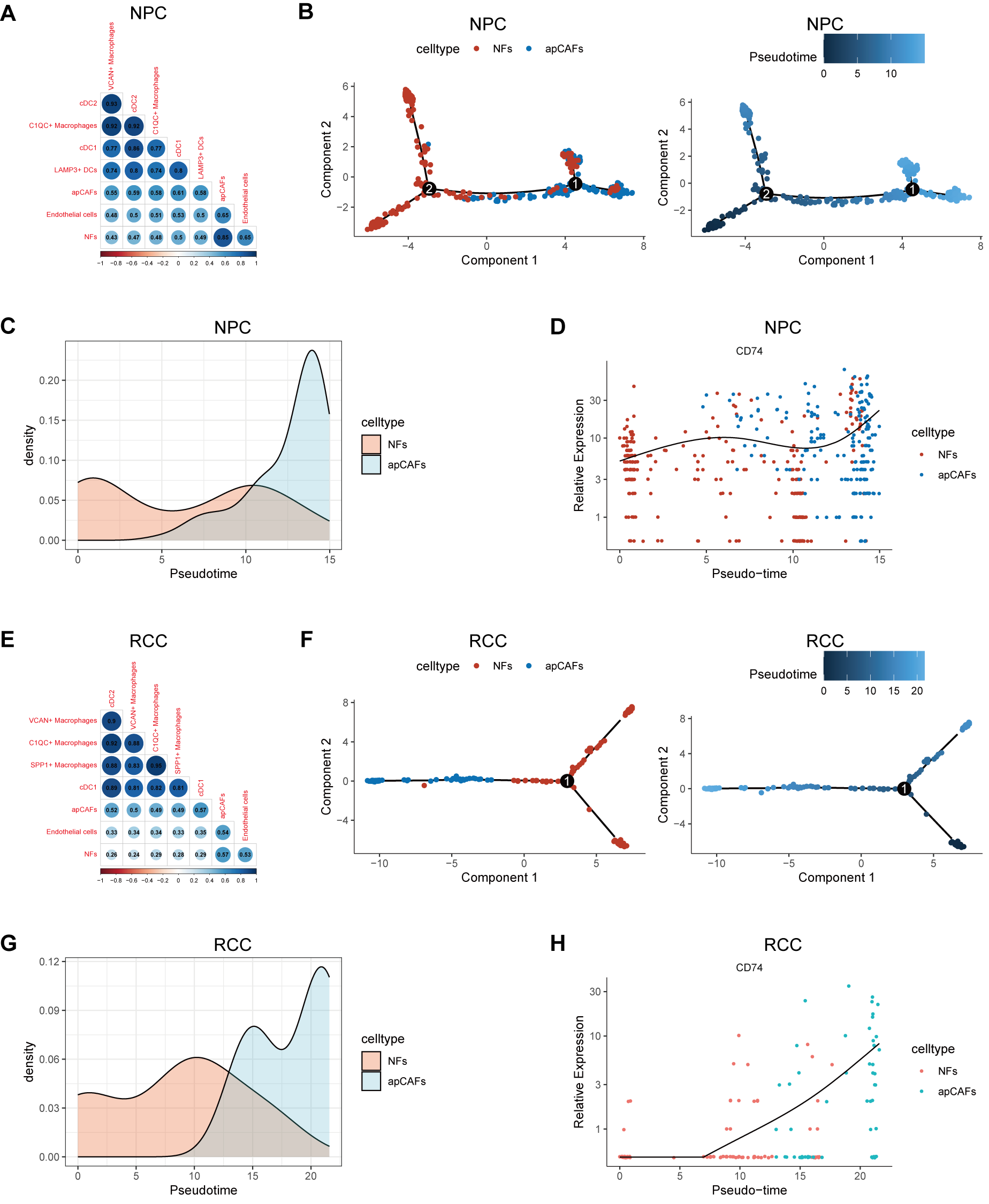

Supplement: Supplementary Figure 14 — Characterization of apCAFs origin. (A, E) Transcriptomic similarity analyses among apCAFs, NFs, endothelial cells, various macrophages and various dendritic cells in NPC (A) and RCC (E). The darker the blue, the stronger the positive correlation, and the darker the red, the stronger the negative correlation. The numbers in the circle represent the correlation coefficient R. (B, F) Left: The cell trajectory along the NFs-apCAFs path in NPC (B) and RCC (F); Right: The pseudotime trajectory along the NFs-apCAFs path in NPC (B) and RCC (F). (C, G) Density distribution of apCAFs and NFs along the pseudotime trajectory in NPC (C) and RCC (G). (D, H) Dynamic variation in CD74 during pseudotime trajectory in NPC (D) and RCC (H). NPC, Nasopharyngeal Carcinoma; RCC, Renal Cell Carcinoma; apCAFs, antigen-presenting CAFs; NFs, normal fibroblasts; cDC1, conventional type 1 dendritic cells; cDC2, conventional type 2 dendritic cells; LAMP3+ DCs, LAMP3+ dendritic cells. [file Image_14.tif]
